# Beyond megadrought and collapse in the Northern Levant: The chronology of Tell Tayinat and two historical inflection episodes, around 4.2ka BP, and following 3.2ka BP

**DOI:** 10.1371/journal.pone.0240799

**Published:** 2020-10-29

**Authors:** Sturt W. Manning, Brita Lorentzen, Lynn Welton, Stephen Batiuk, Timothy P. Harrison

**Affiliations:** 1 Cornell Tree-Ring Laboratory, Department of Classics, Cornell University, Ithaca, NY, United States of America; 2 Department of Near and Middle Eastern Civilizations, University of Toronto, Toronto, ON, Canada; 3 Department of Archaeology, Durham University, Dawson Building, Durham, United Kingdom; Max Planck Institute for the Science of Human History, GERMANY

## Abstract

There has been considerable focus on the main, expansionary, and inter-regionally linked or ‘globalising’ periods in Old World pre- and proto-history, with a focus on identifying, analyzing and dating collapse at the close of these pivotal periods. The end of the Early Bronze Age in the late third millennium BCE and a subsequent ‘intermediate’ or transitional period before the Middle Bronze Age (~2200–1900 BCE), and the end of the Late Bronze Age in the late second millennium BCE and the ensuing period of transformation during the Early Iron Age (~1200–900 BCE), are key examples. Among other issues, climate change is regularly invoked as a cause or factor in both cases. Recent considerations of “collapse” have emphasized the unpredictability and variability of responses during such periods of reorganization and transformation. Yet, a gap in scholarly attention remains in documenting the responses observed at important sites during these ‘transformative’ periods in the Old World region. Tell Tayinat in southeastern Turkey, as a major archaeological site occupied during these two major ‘in between’ periods of transformation, offers a unique case for comparing and contrasting differing responses to change. To enable scholarly assessment of associations between the local trajectory of the site and broader regional narratives, an essential preliminary need is a secure, resolved timeframe for the site. Here we report a large set of radiocarbon data and incorporate the stratigraphic sequence using Bayesian chronological modelling to create a refined timeframe for Tell Tayinat and a secure basis for analysis of the site with respect to its broader regional context and climate history.

## Introduction

Much recent scholarship has focused on identifying apparent periodic episodes of substantial and longer-lasting drought–so-called megadroughts–which arguably appear to correlate, at least approximately in temporal terms, with episodes of major collapse, change, or reorientation in human civilization in various regions of the world [1–3]. Whether mere approximate contemporaneity is indicative of explanation is another question, and recent discussions of social responses to climatic change have highlighted the unpredictability and variability of possible outcomes, precluding a simplistic association between environmental change and societal collapse [4–11]. Nonetheless, chronological resolution is an essential component of consideration of any possible relationships between environmental change and social response [12], and the absence of an appropriate close temporal association undermines many purported causal connections (e.g. [9, 13–15]). The potential importance of such historically relevant climate forcing episodes, both in explaining history, but also as laboratory of current and future relevance, should not be overlooked–and this general topic has been the subject of much recent research, as in other regions, in the Mediterranean-Near East (e.g. [1–3, 16–23]), but also debate (e.g. [4–11, 24]). In several instances, the exact associations and causal connections between observed proxy evidence for climate change and human history have been demonstrated to be both complex and multi-faceted, and often not as clear in temporal or causal terms as sometimes stated by proponents of one or other specific viewpoint (e.g. [9, 14, 15]).

Two multi-century climate change episodes with special relevance to the Levant/East Mediterranean region are regularly cited and stated as occurring from (i) ca. 4200 BP/2200 BCE and (ii) 3200 BP/1200 BCE (BP here from 2000 CE) (e.g. [16–23, 25, 26]). In each instance, there is purportedly a multi-century episode of collapse, then general hiatus and transformation before a subsequent return to ‘high’ civilization—the Middle Bronze Age (the Middle Kingdom in Egypt, the Old Babylonian period in Mesopotamia) from around 2000/1900 BCE for the first, and the Iron Age ‘renaissance’ from the 9-8^th^ centuries BCE for the second (the latter perhaps linked to a major solar irradiance minimum and climate shift [3, 27, 28, ref. 29 pp. 112–115]).

The 2200 BCE episode in the later Early Bronze Age (hereafter EB) is well-dated, both in terms of some wider proxies indicating a shift to cooler and more arid conditions, and in terms of the demise and abandonment of the formerly primate site of Tell Leilan in northern Syria just before 2200 BCE [25, 30, 31]. However, the impact of this climate event is more difficult to reconstruct in the Levant and tightly resolved higher frequency records are sparse. Recent syntheses of radiocarbon (^14^C) evidence have decoupled the chronology of this event from the long-recognized decline in settlement during the EB IV in the southern Levant, which is now recognized to have begun several centuries earlier, ca. 2500 BCE [32, 33]. It is also increasingly evident that sites in the northern Levantine region, which were in less fragile dry-farming loci with significant access to reliable water resources in the form of karstic aquifers, were variably affected and not all collapsed [25, 34, 35]. Weiss indeed argues that the Euphrates and especially Orontes river valleys, and the area of ‘karstic’ springs, formed refugia during the 2200 BCE episode with growth supplemented by habitat-tracking out of other less resilient regions of northern Mesopotamia/Syria [25, 35]. He points to the take-off of Tell Tayinat as an example of this [35]. While the variable ecological context is undoubtedly relevant, other factors also appear to be in play, and not just survival in the face of climate crisis, including the re-orientation and transformation of interregional trade and local economies, and of the relevant trade routes. In particular, these processes saw Anatolia and the East Mediterranean (and so maritime connectivities, versus, or in addition to, land routes) become more important foci for the Levant in the later/late 3^rd^ millennium BCE [36, 37]. In fact, contrary to the pattern observed in areas of northern Mesopotamia, the northern Levant witnesses a persistent tradition of urbanism continuing through and following the period at 2200 BCE [34, 38, 39]. As such, this region offers a key venue for observing alternative trajectories and responses to the 4.2k climate episode.

The 1200 BCE episode, around the close of the Late Bronze Age, involves a long-recognized widespread set of site abandonments, destructions and changes across the East Mediterranean and Levantine region, the collapse of the previous ‘palatial’ economies and inter-linked trading systems and an ensuing ‘dark age’ [11, 15, 24, 40–42]. Associated proxy climate evidence indicates another shift around this period to cooler and more arid conditions [20–23, 26, 43]. The widespread collapse of the internationalized political economies of the Late Bronze Age is real, and many sites do experience destruction, decline, or abandonment. But the collapse of the region is not total. It is increasingly clear that this episode is as much a reorientation and shift to differing social and economic models as simple collapse [11, 36], a reorganization that represents a key formative period that shapes the later developments of the Iron Age. The climate proxy data, while relatively consistent in indicating cooler, more arid conditions, are as yet poorly constrained in temporal terms in many cases, and exact associations with cultural and political developments therefore remain problematic [15].

In each of these cases there remains much to be investigated and learnt in order to be able to write a detailed history successfully and convincingly linking archaeology-history and climate. However, even more pressing is the question: what happened around and particularly *after* these apparent episodes of collapse/change, during the periods of ensuing transformation prior to the generally accepted return of ‘high’ civilization? Despite terms like megadrought and collapse, it is of course widely understood that archaeological and historical evidence continues through these episodes, at least in several areas. We consider here the northern Levantine Early Bronze Age IV as conventionally ca. 2500–2000 BCE (EB IVA ca. 2500–2300; EB IVB ca. 2300–2000 BCE) and the early Iron Age as the period during and after the 12^th^ century BCE in the northern Levant (ca. 1200–900 BCE, Iron Age I). On the principle that both humans and nature abhor a vacuum, we may also assume a corollary if there is a climate association with the changes occurring ca. 2200 BCE and 1200 BCE (or even if there is simply a key structural change in social and economic systems). Thus, if such climate (or other) changes negatively affect one existing set of sites and their environmental and locational contexts, it is also likely that other loci may find an opportunity to fill this void and to offer ‘alternative’ trajectories and histories. These cases might involve societies centered on a different, more resilient, economic base–like pastoralism in the Levant [4, 44], societies constructed on differing socio-political scales (e.g. smaller units), based in less affected geographic contexts, or with access to alternative trade networks [36, 41]. In other words, we open perspective towards both resilience and alternatives to simple collapse, and more nuanced approaches that incorporate a heterogeneity of response and opportunity at local level in the face of regional-global climate processes.

The large and important site of Tell Tayinat in southeast Turkey offers one such alternative history [45–51]. In this paper, we use radiocarbon dates and analysis of these to investigate and define the chronology of the site of Tell Tayinat, and demonstrate that its substantive history of occupation(s) includes the periods of response to the ‘megadrought’ eras associated with both the 2200 BCE and 1200 BCE episodes. The site is largely not occupied during the ‘high-civilization’ periods of the Middle and Late Bronze Age. Instead, the local regional occupation in these periods is at Tell Atchana (ancient Alalakh), and the switch to Atchana from Tayinat at the end of the Early Bronze Age, followed by a return to Tayinat at the end of the Late Bronze Age, forms a local version of the wider ‘alternative’ paradigm of interest [45–51]. Tell Tayinat thus represents an alternative history and trajectory to the ‘palatial’ mainstream of the Levantine region, and a prime case study illustrating how change—whether forced by economics, climate, geography or other factors—is rarely universal. Instead, negative or positive factors affecting one set of circumstances may well have differing and even opposite effects elsewhere. The context and societal formations at Tell Tayinat—and the contrast, locally, with Tell Atchana, and more widely with the major Middle–Late Bronze Age primate sites across the east Mediterranean and Levant—offer us one rich window into a resilient and successful response, and alternative to collapse, in the face of regional megadrought in the Old World.

### Tell Tayinat

**Fig 1:**
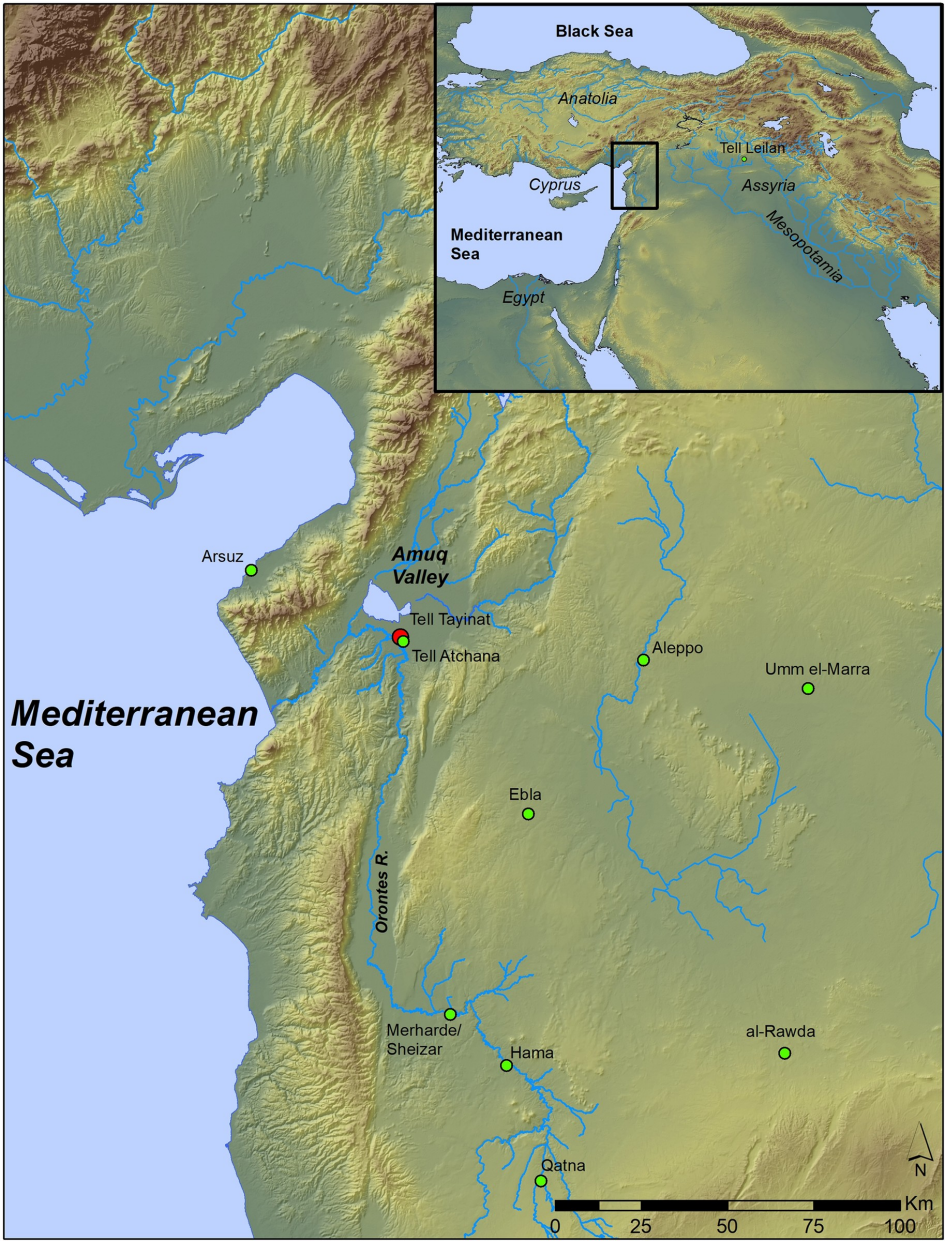
Map showing the Orontes Valley of Northwest Syria and Southeast Anatolia and all the sites discussed in this study. This map was produced in ArcGIS v 10.7.1 at the CRANE funded Archaeology Centre Digital Innovation Laboratory of the University of Toronto by compiling GIS Shapefiles and Digital Elevation Data built from publicly available sources including NASA/JPL/NIMA.

Tell Tayinat, located in the Amuq Plain of southeastern Turkey and centrally positioned in the northern Levantine region, comprises a large, low-lying mound within the flood plain of the Orontes River. Tayinat sits on the northern bend of the Orontes, downstream of its entrance into the Amuq Plain, at the point where it turns westward toward Antakya (ancient Antioch) and the Mediterranean Sea (Fig 1). The site has long been a focus of excavation and study, starting with large-scale excavations conducted by the Oriental Institute of the University of Chicago between 1935 and 1938 [52, 53]. More recently, it has been the focus of work by the Tayinat Archaeological Project (TAP) based at the University of Toronto, which began with pedestrian and geomagnetic survey between 1999–2002 [54], further geomagnetic survey in 2003 (http://sites.utoronto.ca/tap/assets/2003geomagneticsurvey_en.pdf), followed by excavations and study from 2004 onwards [45–51, 55, 56]. The earlier excavations demonstrated that the site was occupied during significant portions of the 3^rd^ millennium BCE, from at least the EB III [52]. To date, the new excavations at the site have produced materials and contexts dating from the later part of the Early Bronze Age (EB IVA-B, late 3^rd^ millennium BCE) and spanning the Iron Age, with significant attention paid to the monumental structures of the Iron II and Iron III levels [53, 57, 58].

Historically, the site is also well-attested. The Early Bronze Age city is perhaps the location referred to as Alalaḫu in records preserved on clay tablets from the late 3^rd^ millennium BCE archive found at the site of Ebla in Syria [49, 50, 59]. For the Iron Age, interpretation of inscriptional evidence has led to suggestions that by at least the 11^th^ century BCE, early Iron Age Tell Tayinat represented the center of a kingdom named Palastin or Walastin, with one king being a certain Taita, “Hero and King of Palastin”, best known from inscriptions in the Aleppo Temple [60–63]. Later historical records, dating to the 9^th^-early 8^th^ centuries BCE, refer to the kingdom as Patina or Unqi [64–66]. The Assyrians took control of the city in 738 BCE and the region became part of their province of Kinalia under an Assyrian governor [65–69].

The end of the first primary period of occupation at Tell Tayinat in the late 3^rd^ millennium BCE corresponds in general with the period of change and re-orientation across much of the Old World from around 4200 years BP or 2200 BCE [16–19, 25]. In the northern Levant, this episode is in general associated with the EB IVB period. The overall dates for the wider period of societal change, commonly associated with a shift(s) in climate affecting especially the Old World region, are largely uncontested—although the exact timings, scale, extent, coherence and uniformity of the climate episode remains debated even in the Mediterranean-Levant region (e.g. [70–73]). Correlating with the end of the Akkadian Empire and the First Intermediate Period in Egypt, a beginning for this period of change in the later 23^rd^ century BCE and especially around 2200 BCE is largely agreed. It is generally accepted that there is a period(s) of apparently cooler and drier conditions in this region that lasts until around the 20^th^ century BCE (ending broadly by around 1900 BCE) [16–19, 25, 30, 31, 70–74, 77, 78]. The area for debate is exactly whether, how, and how comprehensively, the latter causes the former [4, 7–8, 16, 19, 25, 31, 35, 75, 76]. The site of Tell Leilan in northeastern Syria offers a closely resolved date for the beginning of this period [30, 31], as do a variety of proxy climate indicators [25, 70–74, 77, 78]. Its end is marked by the re-emergence of wider trade networks and state-polities (like the Middle Kingdom of Egypt, Old Babylonian Period in Mesopotamia) during the 20^th^ century BCE.

The issue for Tell Tayinat is to position the site and its EB occupation in terms of this wider general timeframe. In contrast to regions such as northern Mesopotamia, whose chronologies have been more closely tied to the 4.2k event, and its apparent historical effects, northern Levantine chronologies generally have not accounted for this event. The general chronological framework for the northern Levant, largely based on sequences from Hama, Ebla and the early Amuq excavations [79], is generally agreed upon, with the EB IVB period conventionally dated to ca. 2300–2000 BCE. More recently, the creation of an EBA-MBA transitional subdivision, ca. 2100–2000 BCE, has been proposed [34, 80, 81]. This chronological framework, however, has been developed primarily from relative ceramic sequences and has not been anchored to ^14^C dates [34, 79, 82]. An independent and direct timeframe is thus lacking. More recently this situation has started to change. ^14^C dates pertaining to several late 3^rd^ millennium BCE sites have been published, including Tell Mardikh/Ebla [83], Rawda [34, 84], Qatna [85] and Umm el-Marra [34]. The suggested date for the destruction of Palace G at Ebla, representing the local transition from the EB IVA to EB IVB, is suggested to date to 2367–2293 BCE, with ca. 54% probability [83], roughly in line with the conventional date for this transition, and placing the 4.2ka (2200 BCE) climate event firmly during the EB IVB period.

The Early Iron Age is more problematic. There has been a long-standing challenge resolving Iron Age chronology in the northern Levant in this period [51, 56, 86, 87]. The close of the Late Bronze Age is characterized by the collapse of the wide-reaching and literate trading systems of previous centuries [15, 40–42, 88–90]. We also lose the well-defined sequence of historical rulers from the unified New Kingdom of Egypt. The result is that archaeologists lack the confident ability to employ replicated material culture associations to tie object types, assemblages, and sites across the eastern Mediterranean to the historical chronology of Egypt. This situation has led to much scholarly uncertainty and debate over dates based on relatively scarce, ambiguous, or contradictory evidence (e.g. [56, 90]). The past couple of decades have, unsurprisingly, seen scholars trying to find alternative means to establish secure chronological timeframes for the early Iron Age in several areas of the East Mediterranean and Aegean, and have–almost inevitably–created controversy as previous hypotheses are challenged, usually by new radiocarbon evidence [86, 87, 91–97].

In the northern Levant, especially, there has been lack of both data and scholarly focus. After the collapse and transformation of the well-known Late Bronze Age civilizations and their major, often ‘palatial’, centers around the close of the 13^th^ century BCE and into the early 12^th^ century BCE [11, 15, 42, 88, 89, 90], the social, political and economic processes of the subsequent earlier Iron Age are much less well understood [51]. This is particularly true in the period stretching from the late 12^th^ through 10^th^ centuries BCE, where historical documentation has been scarce. Although new inscriptional evidence is beginning to provide a historical framework for this period, the precise chronology of these historical developments remains fluid, uncertain, and largely based on paleographical grounds as newly emerging finds regularly require the revision of historical chronologies and king lists [60–62, 98, 99]. In the 9^th^–8^th^ centuries BCE, historical documentation becomes more frequent from Neo-Assyrian records, as a result of their increasing contacts with this region, and then their takeover and administrative control from the late 8^th^ century BCE onward [65–68, 100]. Following the conquest of Kunulua–the ancient name in this period for Tell Tayinat–by the Assyrian ruler Tiglath-pileser III in 738 BCE, Tayinat became the capital of the Assyrian province of Kinalia [58, 64, 65, 101]. The available textual evidence suggests that in the intervening period, the larger political structures of the Late Bronze Age had vanished and in their place was a collection of relatively small territorial states [45, 46, 51, 55, 100–103]. The trajectories and chronologies of these profound changes and of the emergence of these new socio-political formations remains largely unclear. Hence establishing a defined and robust chronology for a key site like Tell Tayinat represents a critical step towards a more nuanced understanding of this time period.

In contrast to the southern Levant and a few other areas, where the widespread use of radiocarbon evidence to address issues of Iron Age chronology has led to the development of a large dataset of chronometric evidence (e.g. [87, 91–97]), available radiocarbon data in the northern Levant is extremely scarce [15, 23, 51, 104]. This has led to the development of a series of relative regional chronologies, often employing widely varying chronological terminologies based on locally-defined or somewhat arbitrary subdivisions [86, 105–108]. These are often based on linkages in material culture and styles within the Levant and with the Aegean and Cyprus [56, 109]. As a result, the issue of chronology has been problematic and largely based on circular reasoning and imprecise criteria.

One of the key discoveries during recent excavations at Tell Tayinat is the articulation of a sequence of early Iron Age remains, after a settlement hiatus during the Middle and Late Bronze Ages, that were unattested in the earlier excavations at the site [45–47, 51, 55, 110]. With an area from ca. 12–20 hectares in extent, Tell Tayinat is one of the larger early Iron Age sites known in the eastern Mediterranean, and represents an important regional sequence for the early Iron Age in the northern Levant. As a result, we report work aimed at remedying the lack of chronological data for this region through establishing a high-resolution calendar timescale for early Iron Age Tell Tayinat. The use of data from Tell Tayinat for the development of a regional sequence is particularly appropriate, given the important role the site played in the early development of radiocarbon dating. A sample of charcoal from Tell Tayinat in fact appeared on Libby’s famous ‘curve of knowns’ as a measurement by the University of Chicago. Libby wrote:

“The next sample, which is marked “Tayinat,” is from a house in Asia Minor which was burned in 675 B.C. It was wood from the floor of a central room in a large *hilani* (“palace”) of the “Syro-Hittite” period in the city of Tayinat in northwest Persia. Its known age is 2625 ± 50 years” [111].

This statement is historic, but also both remarkable and (retrospectively) distressing. A charcoal sample of the scale required at this time for radiocarbon dating likely had a considerable number of tree-rings. Thus it could quite well today have offered a potentially important dendrochronological sample, or least a highly resolved dendro-^14^C-wiggle-match sample. Such a sample could have enabled precise dating (as undertaken at some other Anatolian Bronze and Iron Age cases [78, 112–116]) of some considerable value, perhaps tied to a specific construction episode at the site (its context from the floor in a central room suggests a fallen roof beam). Sadly, no charcoal recovered so far in the recent Tayinat excavations has been of anywhere near such a scale for dendrochronological analysis, and the whereabouts of Libby’s original sample or any others that may have been collected in the 1930s remains unknown. The confidence expressed in the dating of the context and sample from the site is also striking, and almost certainly misplaced, given the complex history of the specific *bīt-hilāni* palace from which the sample was taken (Rooms I-J, 1^st^ floor in Building I) (e.g., [53, 57, 117, 118]).

In view of these research opportunities and the current limitations in terms of dating, our project therefore seeks to integrate the archaeology with the radiocarbon evidence to achieve a secure timeframe for Tell Tayinat in both the late Early Bronze Age and the Early Iron Age. We use organic samples (identified wood charcoal and especially short-lived seed material) from well-defined archaeological contexts at Tell Tayinat for a program of radiocarbon dating. In particular, we have carefully selected sets of short-lived sample material where possible–offering ages contemporary with use–from stratigraphic sequences. These circumstances, where we have an archaeologically-ordered sequence of contexts and samples, allows in addition the application of Bayesian chronological modeling approaches [119–123], where prior archaeological or historical knowledge can be integrated with the radiocarbon probabilities, in order to achieve more precise and more robust chronologies.

## Materials and methods

### Samples and ^14^C dates

We reviewed and identified the available organic sample materials from the Tell Tayinat Archaeological Project (https://tayinat.artsci.utoronto.ca/) and its excavation program. The Tayinat landowners, in particular the Kuseyri family, permitted work on their land. All necessary permits were obtained for the described study, which complied with all relevant regulations. The Directorate of Cultural Heritage and Museums of Turkey granted the research permits necessary to conduct each of the Tell Tayinat excavation seasons. All fragments of wood charcoal chosen for examination that were larger than 2 mm were fractured by hand or with a steel razor blade to create fresh transverse, radial, and tangential planes, in order to examine the wood anatomical structure. After fracturing, samples were supported in a sand bath and examined under a Motic K-400P stereo microscope at x6 to x50 magnification and an Olympus BX51 polarizing microscope at x50 to x500 magnification. The micro-anatomical features of each section were documented, photographed, and compared with those from modern reference collection materials, standard reference texts [124, 125], and the InsideWood online database (https://insidewood.lib.ncsu.edu/). Seed and non-wood botanical remains were identified by comparing sample morphological characteristics with modern reference materials and reference seed atlases (e.g., [126, 127]). A LEO 1550 field emission scanning electron microscope (FESEM) was used for high magnification observation of plant micro-features and high-quality image capture.

Following identification, we selected a number of samples (both wood charcoal and short-lived seeds) for which reasonably secure archaeological associations are available (as is inevitable at a complicated multi-period tell site with such a multi-phase stratigraphic sequence, the work of this project has in fact led to the reassessment of a few contexts–see below). The general Tell Tayinat site stratigraphic sequence is set out in Table 1; further stratigraphic description and the discussion of associated material culture can be found in [48, 50, 51, 53, 110, 118]. The samples ultimately selected for ^14^C dating and any comments related to their archaeological contexts are listed in Table 2. These samples were then radiocarbon dated at the Oxford Radiocarbon Accelerator Unit. Acid-Base-Acid (ABA) sample pretreatment, target preparation, and Accelerator Mass Spectrometry (AMS) ^14^C dating were performed following methods described previously [128–130]. Isotopic fractionation has been corrected for employing the δ^13^C values measured on the AMS–the quoted δ^13^C values were measured independently on a stable isotope mass spectrometer (±0.3‰ relative to VPDB). The 49 new ^14^C dates acquired are listed in Table 3.

**Table 1:** The Tell Tayinat general stratigraphic sequence indicating contexts of radiocarbon dated samples. Descriptions of associated stratigraphy and material culture can be found in [48, 50, 51, 53, 110, 118]. The conventional dates for the associated periods are indicated following [105–106] for the Iron Age and [34] for the Early Bronze Age, while previously published dates for the main Tayinat phases are indicated in parentheses and italics following [53].

General Site Phases	Historical Periods [34, 105–106] and Previous Dates [53]	Field 1	Field 2	Field 3	Field 7	Comments
Modern	Modern	1	1a/b	1	1	Modern topsoil and disturbances. Includes Chicago trenches
			1c			
1	Iron III, ca. 738–600 BCE, *(720–680 BCE)*	2a	2a	2a?	2a	Field 1: renovation, reuse Temple II [53, 118]; Field 2: renovation, reuse Building XVI [118]
						No samples analyzed
2 Late 2	Iron II, ca. 900–738 BCE (*825–720 BCE)*	2b	2b1-2	2b	2b	Field 1: earliest phase of Temple II? [53, 118]; Field 2: fill immediately above stone paving outside Building XVI [118]
		OxA-30320	OxA-32164, 32166, 32167, 32168, 32169	OxA-30309		
2 Late 1		GAP?	2b3		2c	Field 2: earliest phase of Building XVI, stone paving [118]
			OxA- 32165 30312?			
2 Middle B		GAP?	3	3a?	3	Field 2: Sounding below Building XVI [118]
			OxA-30321		OxA-30315	
2 Middle A(2)		2c	?			Field 1: Infill of ditch/street [118]
		OxA-32170, 32171, 32172				
2 Middle A(1) and BP1	Iron I-II trans.	2d	4a	3b?	4	Field 1: Ditch and sherd paved street [118]; Field 2: Chicago Building Period 1, Building XIV [53, 118]
2 Early	Iron I, ca. 1200–900 BCE *(875–825 BCE)*	GAP	4b-5a-5b	4?	5	Field 2: Domestic occupation cut by foundations of Building XIV [118]
			OxA-30322, 30318			
3		3	6		6	Field 1: Ephemeral occupation, primarily pits [51]
4		4		5?		Field 1: Major architectural phase, domestic? [51]
5a		5a	7			Field 1: Domestic occupation [51]
		OxA-30324, 30329, 30563, 30565				
5b		5b				Field 1: Domestic occupation [51]
		OxA-30310, 30311, 30324, 30563, 30565, 32141, 32142, 32143, 32162, 32163				
6a		6a				Field 1: Domestic occupation [51]
		OxA-30314, 30319, 30326, 30327, 30328, 30421				
6b		6b				Field 1: Earliest IA architecture, domestic [51, 110]
		OxA-30317, 30323, 30421, 30564, 32140, 32139				
6c		6c				Field 1: Earliest IA re-occupation, no architecture, primarily pits [51, 110]
		OxA-30313				
*GAP*	*Late Bronze Age*	*GAP*				*No occupation*
*GAP*	*Middle Bronze Age*	*GAP*				*No occupation*
7	EBIVB, ca. 2300-2100/2000 BC	7	8–9			Field 1: Ephemeral terminal EB occupation. No architecture [48, 50]
		OxA-30325, 32134, 32135, 32136, 32137, 32138, 32347				
8a		8a				Field 1: Debris associated with destruction of FP8b structure [48, 50]
		OxA-32132, 32133				
8b		8b				Field 1: Construction of large structure [48, 50]
		OxA-30316				
9	EBIVA-B, ca. 2300 BC	9				Field 1: Short intermediate phase between construction phases [48, 50]
10	EBIVA, ca. 2500–2300 BC	10				Field 1: Large structure, evidence of destruction. Excavated 2017

**Table 2:** Samples for ^14^C dating from Tell Tayinat obtained by this project. For comments on some of the sample contexts, see the notes below the table. Tayinat General Period Scheme refers to the General Site Phases as identified in Table 1. Local Field Phase refers to the individual phasing schemes devised independently for each excavation area, outlined in Table 1.

Laboratory ID, OxA-	Sample ID	Area	Field. Square	Locus	Pail	Period	Local Field Phase (FP)	Tayinat General Period Scheme	Sample
30309	SA1238	3	H3.77	21	45	Iron II?	2b	2 Late 2	*Olea europaea* pit
30310	SA1204	1	G4.56	135	265	Iron I	5b	5b	*Olea europaea* pit
30311	SA1204	1	G4.56	135	265	Iron I	5b	5b	*Olea europaea* pit
30312	SA4778	2	G4.38	8	75	Iron II/III?	2b3	2 Late 1^a^	*Olea europaea* pit
30313	SA6449	1	G4.56	246	548	Iron I	6c	6c	*Quercus* sp.
30314	SA3329	1	G4.66	0^b^	0^b^	Iron I	6a	6a	*Quercus* section *Cerris*
30315	SA7829	7	G4.58	16	59	Iron II	3?	2 Middle B	Salicaceae
30316	SA6442	1	G4.55	258	604	EB IVb	8b	8b	*Pinus brutia*
30317	SA4791	1	G4.56	194	380	Iron I	6b	6b	*Tamarix* sp.
30318	SA1683	2	G4.46	11	37	Iron I	5a	2 Early	*Vitis vinifera* pip
30319	SA4793	1	G4.56	196	386	Iron I	6a?	6a?	Betulaceae cf. *Ostrya carpinifolia*
30320	SA4205	1	G4.56	188	367	Iron I	6b, but intrusive from 2b	Reassigned to 2 Late 2	*Cicer arietinum* seed
30321	SA7839	2	G4.48	38	130	Iron II	3	2 Middle B	Bark
30322	SA792	2	G4.35	18	66	Iron I	5b	2 Early	*Pinus brutia*
30323	SA4749	1	G4.56	194	381	Iron I	6b	6b	*Tamarix* sp.
30324	SA1202	1	G4.56	112	245	Iron I	5a/b	5a/b	Betulaceae
30325	SA3091	1	G4.55	154	298	EB IVb	7	7	*Pinus brutia*
30326	SA1236	1	G4.56	127	225	Iron I	6a	6a	*Fraxinus* sp.
30327	SA3959	1	G4.56	174	330	Iron I	6a	6a	Betulaceae cf. *Ostrya carpinifolia*
30328	SA3959	1	G4.56	174	330	Iron I	6a	6a	Betulaceae cf. *Ostrya carpinifolia*
30329	SA1200	1	G4.56	98	170	Iron I	5a?	5a?	Evergreen *Quercus*
30421	SA4198	1	G4.56	181	359	Iron I	6a/b?	6a/b?	*Rhamnus* / *Phillyrea*
30563	SA1199	1	G4.56	112	177	Iron I	5a/b	5a/b	*Olea europaea* pit
30564	SA4805	1	G4.56	206	410	Iron I	6b	6b	*Pinus brutia*
30565	SA1210	1	G4.56	112	245	Iron I	5a/b	5a/b	Deciduous *Quercus*
32132	SA5307	1	G4.55	271	496	EB IVb	8a	8a	*Olea europaea* pit
32133	SA5339	1	G4.55	271	507	EB IVb	8a	8a	*Olea europaea* pit
32134	SA3977	1	G4.55	216	369	EB IVb	7	7	*Olea europaea* pit
32135	SA6479	1	G4.56	252	559	EB IVb	7	7	*Olea europaea* pit
32136	SA6533	1	G4.56	270	601	EB IVb	7	7	*Olea europaea* pit
32137	SA6466	1	G4.56	249	554	EB IVb	7	7	*Olea europaea* pit
32138	SA6497	1	G4.56	261	578	EB IVb	7	7	*Olea europaea* pit
32139	SA5113	1	G4.56	214	435	Iron I	6b (but residual?)	6b (but residual?)	*Olea europaea* pit
32140	SA5533	1	G4.56	232	494	Iron I	6b, but intrusive from 5b	Reassigned to 5b	*Olea europaea* pit
32141	SA1973	1	G4.56	119	226	Iron I	5b	5b	*Olea europaea* pit
32142	SA1974	1	G4.56	143	271	Iron I	5b	5b	*Olea europaea* pit
32143	SA1975	1	G4.56	135	268	Iron I	5b	5b	*Olea europaea* pit
32162	SA1976	1	G4.56	138	275	Iron I	5b	5b	*Olea europaea* pit
32163	SA1976	1	G4.56	138	275	Iron I	5b	5b	*Olea europaea* pit
32164	SA5076	2	G4.37	7	23	Iron II/III?	2b1	2 Late 2	*Olea europaea* pit
32165	SA5311	2	G4.37	7	37	Iron II/III?	2b1 (but residual?)	2 Late 1^a^	*Olea europaea* pit
32166	SA5313	2	G4.48	27	95	Iron II/III?	1c^C^	2 Late 2	*Olea europaea* pit
32167	SA5458	2	G4.28	6	45	Iron II/III?	2b1	2 Late 2	*Olea europaea* pit
32168	SA2859	2	G4.47	5	37	Iron II/III?	2b1	2 Late 2	*Olea europaea* pit
32169	SA4777	2	G4.38	11	76	Iron II/III?	2b1	2 Late 2	*Olea europaea* pit
32170	SA2309	1	G4.66	81	204	Iron II	2c	2 Middle A2	*Olea europaea* pit
32171	SA2306	1	G4.65	94	244	Iron II	2c	2 Middle A2	*Olea europaea* pit
32172	SA2907	1	G4.66	88	228	Iron II	2c	2 Middle A2	*Olea europaea* pit
32347	SA3975	1	G4.55	232	393	EB IVb	7	7	*Olea europaea* pit

^a^ SA4778 was from the surface of the central room of Temple XVI, whose latest use phase should be Iron III based on the historically dated tablet found within it [68]. However, we regard this sample and also SA5311 as likely residual material belonging to the earlier use of this temple space and not from its very last phase of use. Hence these samples are assigned to Phase 2 Late 1.

^b^ This sample (outer rings) was from a larger wood sample extracted from the balk; hence it is designated as Locus 0, but it is equivalent to G4.66 Locus 33 and hence Phase 6a.

^c^ This sample was excavated from a locus identified as fill from the Chicago excavation trench and hence was assigned to FP1c (modern); however, this sample lay immediately above the stone paving and produces a date consistent with other samples from the same context (Phase 2 Late 2), hence for modelling purposes we treat it as Phase 2 Late 2 in the Tayinat general sequence.

**Table 3:** ^14^C dates on the samples in Table 2.

Laboratory ID	Sample ID	Tayinat General Period Scheme	Sample	^14^C Age ± 1σ	δ^13^C‰
				(years BP)	
OxA-30309	SA1238	2 Late 2	*Olea europaea* pit	2519±26	-21.8
OxA-30310	SA1204	5b	*Olea europaea* pit	2810±28	-23.4
OxA-30311	SA1204	5b	*Olea europaea* pit	2806±31	-23.1
OxA-30312	SA4778	2 Late 1	*Olea europaea* pit	2679±28	-20.9
OxA-30313	SA6449	6c	*Quercus* sp.	3038±28	-25.9
OxA-30314	SA3329	6a	*Quercus* section *Cerris*	2948±26	-25.8
OxA-30315	SA7829	2 Middle B	Salicaceae	2679±27	-30.0
OxA-30316	SA6442	8b	*Pinus brutia*	4048±29	-23.4
OxA-30317	SA4791	6b	*Tamarix* sp.	2962±27	-26.2
OxA-30318	SA1683	2 Early	*Vitis vinifera* pip	2837±27	-26.5
OxA-30319	SA4793	6a?	Betulaceae cf. *Ostrya carpinifolia*	2918±27	-26.8
OxA-30320	SA4205	Reassigned to 2 Late 2	*Cicer arietinum* seed	2546±27	-24.6
OxA-30321	SA7839	2 Middle B	Bark	2739±26	-24.6
OxA-30322	SA792	2 Early	*Pinus brutia*	3047±26	-24.0
OxA-30323	SA4749	6b	*Tamarix* sp.	2929±28	-26.6
OxA-30324	SA1202	5a/b	Betulaceae	2821±26	-26.6
OxA-30325	SA3091	7	*Pinus brutia*	3871±29	-24.1
OxA-30326	SA1236	6a	*Fraxinus* sp.	2808±29	-25.7
OxA-30327	SA3959	6a	Betulaceae cf. *Ostrya carpinifolia*	2896±26	-25.2
OxA-30328	SA3959	6a	Betulaceae cf. *Ostrya carpinifolia*	2891±27	-25.4
OxA-30329	SA1200	5a?	Evergreen *Quercus*	2829±27	-23.7
OxA-30421	SA4198	6a/b?	*Rhamnus*/*Phillyrea*	2872±31	-23.4
OxA-30563	SA1199	5a/b	*Olea europaea* pit	2857±27	-23.6
OxA-30564	SA4805	6b	*Pinus brutia*	2882±26	-24.0
OxA-30565	SA1210	5a/b	Deciduous *Quercus*	2861±27	-24.7
OxA-32132	SA5307	8a	*Olea europaea* pit	3861±31	-20.2
OxA-32133	SA5339	8a	*Olea europaea* pit	3799±28	-20.1
OxA-32134	SA3977	7	*Olea europaea* pit	3737±29	-22.5
OxA-32135	SA6479	7	*Olea europaea* pit	3784±30	-22.3
OxA-32136	SA6533	7	*Olea europaea* pit	3830±29	-21.4
OxA-32137	SA6466	7	*Olea europaea* pit	3765±30	-22.1
OxA-32138	SA6497	7	*Olea europaea* pit	3697±29	-20.6
OxA-32139	SA5113	6b (but residual?)	*Olea europaea* pit	3717±30	-21.8
OxA-32140	SA5533	6b (reassigned to 5b)	*Olea europaea* pit	2806±30	-21.4
OxA-32141	SA1973	5b	*Olea europaea* pit	2886±28	-21.2
OxA-32142	SA1974	5b	*Olea europaea* pit	2805±30	-21.2
OxA-32143	SA1975	5b	*Olea europaea* pit	2786±29	-22.3
OxA-32162	SA1976	5b	*Olea europaea* pit	2839±26	-20.6
OxA-32163	SA1976	5b	*Olea europaea* pit	2811±27	-20.7
OxA-32164	SA5076	2 Late 2	*Olea europaea* pit	2516±26	-23.2
OxA-32165	SA5311	2 Late 1	*Olea europaea* pit	2732±27	-21.7
OxA-32166	SA5313	2 Late 2	*Olea europaea* pit	2506±25	-20.8
OxA-32167	SA5458	2 Late 2	*Olea europaea* pit	2545±25	-23.2
OxA-32168	SA2859	2 Late 2	*Olea europaea* pit	2511±25	-21.8
OxA-32169	SA4777	2 Late 2	*Olea europaea* pit	2486±26	-22.1
OxA-32170	SA2309	2 Middle A2	*Olea europaea* pit	2814±26	-21.8
OxA-32171	SA2306	2 Middle A2	*Olea europaea* pit	2784±27	-19.7
OxA-32172	SA2907	2 Middle A2	*Olea europaea* pit	2798±27	-20.9
OxA-32347	SA3975	7	*Olea europaea* pit	3772±26	-19.5

### Bayesian chronological modeling

In order to best estimate and quantify the calendar age ranges for the Tayinat archaeological phases we employed Bayesian Chronological Modeling [119–123, 131], employing OxCal 4.3.2 software [119, 121, 132] (https://c14.arch.ox.ac.uk/oxcal.html), in order to integrate prior archaeological sequence information with the radiocarbon dating probabilities from the measured samples (Tables 1–3). OxCal terms such as “Sequence”, “Phase”, and “Boundary”, are capitalized in the text and figures below. We employ the current revised northern hemisphere radiocarbon calibration dataset, IntCal20 [133]. The results for the periods under investigation are only slightly different when compared with the previous IntCal13 dataset [134] and we compare the results for Model 2 below. We also briefly discuss the issue of the potential relevance of a small Mediterranean region growing-season offset for high-precision ^14^C age calibration (see below).

Where possible, we sought to employ short-lived (annual) samples for dating contexts, since these samples–if they are in their primary stratigraphic context related to human use–offer dates directly relevant to the contexts of discovery. Within OxCal, we tested the coherence of the short-lived samples with the model using the General Outlier model of OxCal [135]—labelled as “SL”, for short-lived, in the OxCal runfiles in the S1 File—in which a Posterior value is calculated for each dated element versus the acceptable Prior value of 5 (that is: a 5% probability of being an outlier). We also consider the OxCal Agreement value for each individual sample (the approximate satisfactory value is 60) and for the overall model (A_model_ and A_overall_ values–again the satisfactory level is about 60). It is important to stress that each run of complicated OxCal models achieves very slightly different results, although for well-constrained model elements within such models, results typically do not vary by more than zero to a couple of years. We quote typical examples where the model converged successfully for the dated elements (Convergence, C, values of 95 or greater).

We also included a number of wood charcoal samples. These introduce issues of in-built age, which we tried to minimize by selecting (when possible) samples from juvenile stems and branches, and shorter-lived species. The expectation is that from a random population, some dated wood will be older (even much older) than the find context, but many samples will only be a little older to around about the contemporary age (whether outer rings, young trees, or branches/twigs), but with a little noise. To try to allow for and to compensate for this, we employed the Charcoal Plus Outlier model in OxCal [136, 137], allowing us to better estimate the date at which groups of charcoal samples from a context were actually used by humans, especially when information from dates on short-lived samples could also be incorporated within the relevant phase grouping. (Note: to use the Charcoal Plus Outlier model, an OxCal.prior file must first be loaded–we list the relevant file for use as /IA.prior in S1 File.) For discussion and illustration of how the use of the Charcoal Plus Outlier model (or the standard Charcoal Outlier model in OxCal [135]) is key to achieving a plausible age model for Tell Tayinat, integrating both the *terminus post quem* (TPQ) information (with varying lengths of the “post” in the *terminus post quem*) from long-lived charcoal samples with the contemporary age information provided by dates on short-lived samples (where they are in the correct context associations), see S2 File (and compare shifts to more recent age ranges for the wood-charcoal samples shown in S1–S3 Figs). We employ the Charcoal Plus Outlier model as more appropriate for the reasons outlined in the studies cited [136, 137], however, very similar results are obtained with the OxCal Charcoal Outlier model [135], as we illustrate in the case of our Model 2, see S3 File. Our stated calendar date ranges are thus estimates of the dated periods or episodes but, where a range of charcoal samples alone are involved, these likely still include some aspect of a *terminus post quem* (TPQ) range, and thus might be described as estimates of a close TPQ and/or the date range. Where the identical sample was dated twice we combined the two measurements into a single weighted average value [138].

Four samples require comment. Sample SA4205 (date OxA-30320) was recorded as from a Phase 6b (Iron Age I) context, but is clearly an intrusive Iron Age II sample as evident from the ^14^C age. After re-examination of the excavation records, it was determined to be intrusive from the immediately overlying foundations of a late Iron II building (Building II) that were cutting into these FP6b levels, and is therefore (re-)assigned to Phase 2 Late 2. This datum is shown in purple in the models below to show it was reassigned. Sample SA5311 (date OxA-32165) came from a Phase 2 Late 2 context, but its ^14^C age is a little older than the other Phase 2 Late 2 samples. However, based on examination of site records, we believe that, like SA4778 (OxA-30312), this sample in fact comes from earlier activity within Phase 2 Late overall—Phase 2 Late 1—versus the final use of this area in the subsequent Phase 2 Late 2 and so is residual material in terms of Phase 2 Late 2. We have thus reassigned the sample to Phase 2 Late 1. It is shown in purple in the models below to indicate that it was residual and reassigned. Sample SA5113 (date OxA-32139) yields a ^14^C age that is around 1000 years older than the other short-lived samples from its supposed Phase 6b context. This situation likely indicates that it is, unrecognized at the time of excavation, (highly) residual material, likely originating from underlying EB IVB layers. It is thus shown in orange in the models below. Finally, Sample SA5533 (date OxA-32140), which was assigned to FP6b, was identified as an outlier in this phase. Upon re-examination of the field records, it was determined that the area in which this sample was excavated was noted at the time of excavation as belonging to a later intrusive pit cutting FP6b levels, and which was assigned a new locus number the following day. This sample should therefore be considered to originate from pit locus 233/234, and has thus been re-assigned to FP5b for modelling purposes.

We built an initial model incorporating all the data available and the stratigraphic information summarized in the Tayinat General Period Scheme in Table 1: Model 1. We explicitly do not make historical assumptions. Thus we do not assume that the transition from Phase 2 to Phase 1 marks the Assyrian conquest in 738 BCE—rather, we run the models with the ^14^C data and stratigraphic knowledge we have independently, and then consider where an event like the Assyrian conquest likely occurred in terms of the site sequence. Without specific confirmatory evidence, it is dangerous (and often circular) in practice and in theory to assume an historical association with any particular destruction or stratigraphic change [139, 140]. Similarly, we also make no assumptions based on existing dates derived from assessments from relative chronologies. As noted above, these are largely based on flexible or circular reasoning. Even in recent historic periods, uncritical dating based on artefact presence/absence is fraught with problems around availability, consumer choice, curation, time-lags and the nature of disposal, and can easily end up being misleading (e.g. [141, 142]). We instead employ the stratigraphic sequence and then let the ^14^C data directly describe the temporal scale.

There are two exceptions to the preceding general statements. First, although we do not assume that the transition from Phase 2 to Phase 1 is coeval with the Assyrian conquest of 738 BCE, it is nonetheless historically attested knowledge that after 738 BCE the Assyrians ruled at Tayinat (Kunulua). Thus it is reasonable to regard some part of the final phase at Tayinat as dating after 738 BCE; hence we use an After command within Phase 1 (Iron III) where we have no ^14^C data. The second element of historical knowledge is that because of the presence of a text with Esarhaddon’s adê of 672 BCE inside a Phase 1 temple at Tayinat [65, 68, 143], we may regard the end of the Tayinat occupation sequence as at least after 672 BCE—again we employ an After command in the OxCal code. In reality, it is possible that occupation at Tayinat continued for some decades (or more) after 672 BCE, maybe towards ~600 BCE. However, we currently lack any ^14^C data or other secure historical date. Thus the dating model ends at present more or less immediately after 672 BCE for lack of any quantifiable information.

The available stratigraphic information pertains only to the level of the site Phases recognized (see Table 1 for current Tell Tayinat relative stratigraphic sequence). Within each Phase we adopt the conservative assumption that the samples are random samples from a uniform distribution, and so could come from any point within the Phase with equal probability: a uniform prior assumption. An obvious exception are the two dates on short-lived samples (olive pits) from the period 8a “destruction event”, which we might assume to lie at the close of Phase 8a. However, these are the only data for Phase 8a (so we lack a ‘distribution’)–hence we use the weighted average value of these two dates as the date of the 8a destruction event. One of the short-lived samples in our dataset, OxA-32139 (sample SA5113), noted above, stands out as a complete outlier with the General Outlier model in OxCal [135] with a Prior of (the maximum value) 100 > Posterior value of 5 (and an OxCal Agreement value, A, less than 6, well below the satisfactory threshold value of >60). This sample, found in a Phase 6b context, is (as noted above) approximately 1000 years older and would likely seem to be a residual sample from the known underlying Early Bronze IV strata in this excavation unit. Notably OxA-30320 and OxA-32140, which we re-assigned (see above), and the ‘problematic’ sample just noted (OxA-32139)—all unrecognized at the time of excavation—come from period 6b and from field square G4.56 in Area 1. One is clearly residual from earlier layers (OxA-32139), one is clearly intrusive from later levels (OxA-30320), and one was confirmed as intrusive from (somewhat later) FP5b levels based on excavation records (OxA-32140). This reflects the challenging nature of the excavation contexts in this excavation square, which displayed frequent pitting activities and some disturbance from the foundations of later Iron II constructions. Most interestingly, as regards associations with regional climate change history and environmental contexts, the Tell Tayinat occupation sequence, as currently represented by the TAP excavations, includes EB IVB levels, followed by reoccupation spanning the Iron Age I-III, with no intervening Middle or Late Bronze Age contexts (see above). Thus residual EBA material in an otherwise Iron Age context is possible, due to the absence of intervening strata. A few instances of somewhat later Iron Age materials occurring as intrusives into early Iron Age contexts are a predictable issue of concern in a multi-period excavation like Tell Tayinat, particularly in an area cut by the foundations of later Iron Age constructions. We exclude OxA-32139 from the remainder of our analyses. The revised model (minus OxA-32139), Model 2, was then employed for the site dating. OxCal runfiles for Model 1 and Model 2 are listed in S1 File.

## Results

**Fig 2:**
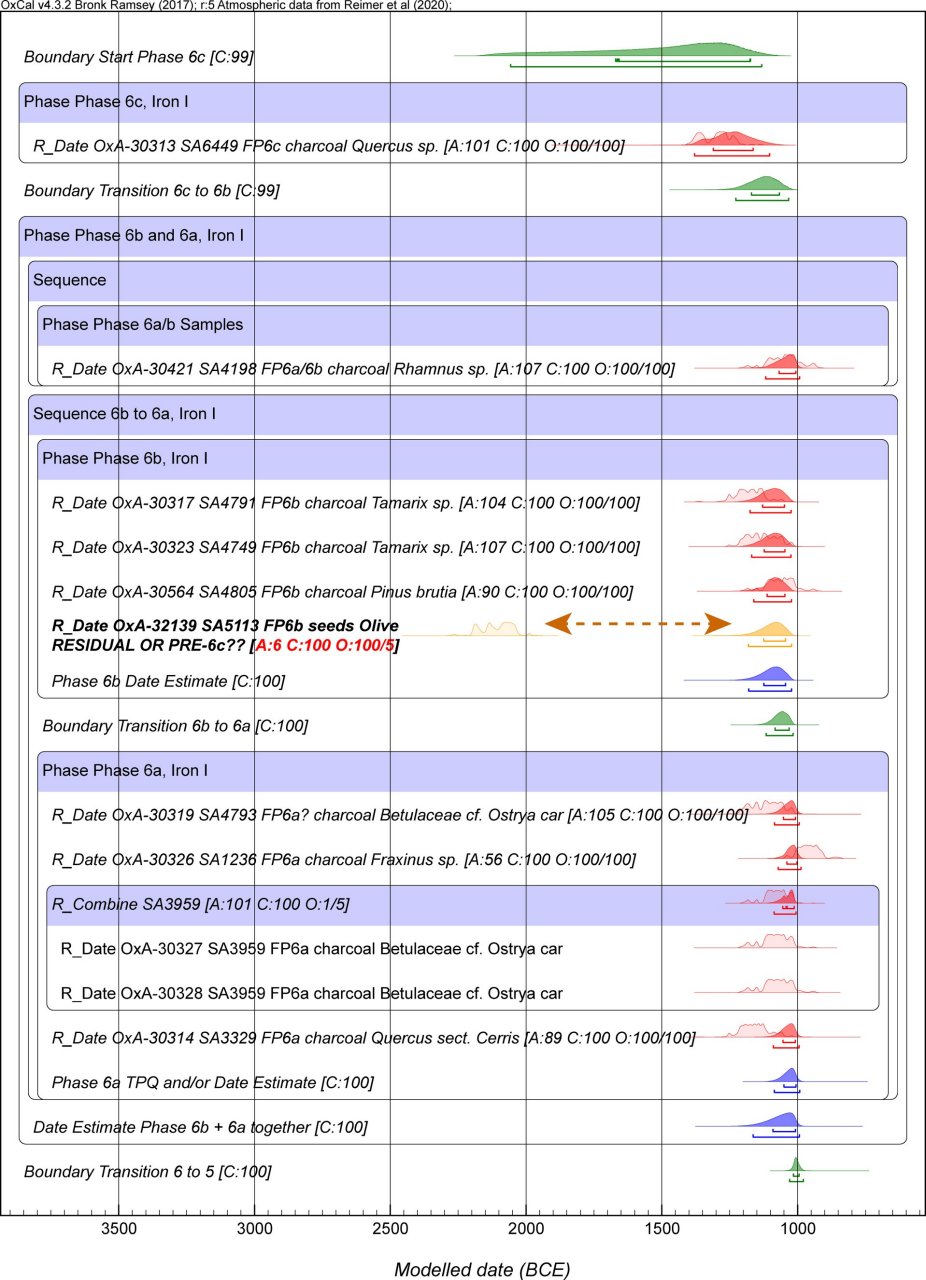
The portion of Model 1 showing the period 6 data to illustrate the very large outlier OxA-32139 on sample SA5113 (see text). This sample is excluded from the remainder of the modeling in our study. Data from OxCal 4.3.2 and IntCal20 with calibration curve resolution set at 1 year. The OxCal Agreement (A) values, the Posterior v. Prior values from the OxCal General Outlier model (O) for the short-lived samples, and the Convergence (C) values are all shown. The dates on wood charcoal samples with the Charcoal Outlier Plus model applied always have an outlier value of 100/100. The light-shaded red probability distributions for each dated sample are the non-modeled calibrated age probability distributions for each sample in isolation. The dark red probability distributions are the modeled (posterior density) calendar age probability distributions. The lines under each probability distribution indicate the modeled 68.2% and 95.4% highest posterior density (hpd) ranges. (Note: OxCal from version 4.4.1 uses 68.3% hpd ranges, however, since we employed OxCal 4.3.2 in this paper, we list 68.2% ranges following OxCal version 4.3.2.).

We first considered Model 1, which employs all the data, and then the very slightly revised Model 2 removing the outlier, OxA-32139, as noted above. Model 1 does not quite achieve satisfactory OxCal A_model_ and A_overall_ values given the outlier just noted (typically ~59–60), with one very low individual OxCal Agreement value (<6) for OxA-32139. The Model 1 run values quoted in Table 4 and the model run shown in S1 and S2 Figs achieved satisfactory Convergence (C) values ≥95 for all elements. However, it should be noted that, especially without use of a high initial kIterations value, runs of Model 1 in a number of cases fail to achieve ≥95 Convergence values for some of the late elements in the model (Phase 2 Late 1 onwards). This problem usually does not occur once OxA-32139 is excluded in Model 2. Fig 2 shows the Phase 6 part of Model 1 and indicates the extreme outlier date of OxA-32139 on sample SA5113. For the full Model 1, see S1 File, S1 and S2 Figs. The placement of the Model 1 dated elements versus IntCal20 (and with the previous IntCal13 shown for comparison) is shown in S3 Fig.

**Table 4:** Selected modeled calendar age ranges from the models and outputs shown in Figs 3–5. **TPQ refers to a date solely from a long-lived sample(s).** Typical results shown; very small variations (often of around 1 year) occur between different model runs (we illustrate by giving results from a very similar but different model run in S3 File, where a number of start/end dates for some of the ranges are 1 year different). For comparison of the results for Model 2 with the previous IntCal13 [134], see below in Table 6. Whole ranges only are listed (compare with S3 File where sub-ranges are listed). The Time Span Phases 4&3 Date estimate combines the separate start (Phase 4) and end (Phase 3) Date estimates in the model.

	Model 1 with all data,		Model 2 *excluding* OxA-32139,	
	A_model_ ~59, A_overall_ ~60		A_model_ ~81, A_overall_ ~80–81	
	*68*.*2% hpd*	*95*.*4% hpd*	*68*.*2% hpd*	*95*.*4% hpd*
	*Date BCE*	*Date BCE*	*Date BCE*	*Date BCE*
**Phase 8b EB IVB TPQ**	2518–2330	2585–2243	2517–2331	2585–2245
**Phase 8a, EBIVB Destruction Event**	2335–2211	2397–2202	2335–2211	2396–2202
**Phase 7 Date Estimate**	2219–2140	2282–2074	2219–2140	2281–2074
**Boundary End Phase 7**	2186–2104	2200–2005	2187–2104	2199–2006
**Phase 6c, Iron I TPQ**	1311–1164	1380–1103	1309–1159	1379–1101
**Phase 6b Date Estimate**	1124–1045	1181–1023	1122–1045	1176–1023
**Phase 6a TPQ and/or Date**	1050–1006	1086–993	1052–1006	1089–992
**Phase 5b Date Estimate**	1008–987	1019–970	1008–987	1019–970
**Phase 5a Date Estimate**	999–975	1006–952	998–976	1006–952
**Time Span Phases 4&3 –No Samples**	987–951	997–921	987–951	997–920
**Phase 2 Early Date Estimate**	970–931	982–894	971–931	982–894
**Phase BP1, Chicago, and Phase 2 Middle A1 –No Data**	955–911	961–866	955–911	961–866
**Phase 2 Middle A2 Date Estimate**	925–855	933–841	926–855	933–841
**Phase 2 Middle B Date Estimate**	900–839	910–828	900–839	910–828
**Phase 2 Late 1 Date Estimate**	836–782	868–766	836–782	868–766
**Phase 2 Late 2 Date Estimate**	772–753	793–733	772–753	791–735
**Boundary Transition Phase 2 to 1**	764–743	771–672	764–743	771–721
**Assyrian Conquest**	738	738	738	738
**Boundary End Tayinat Sequence**	672–669	674–668	672–669	674–668

**Fig 3:**
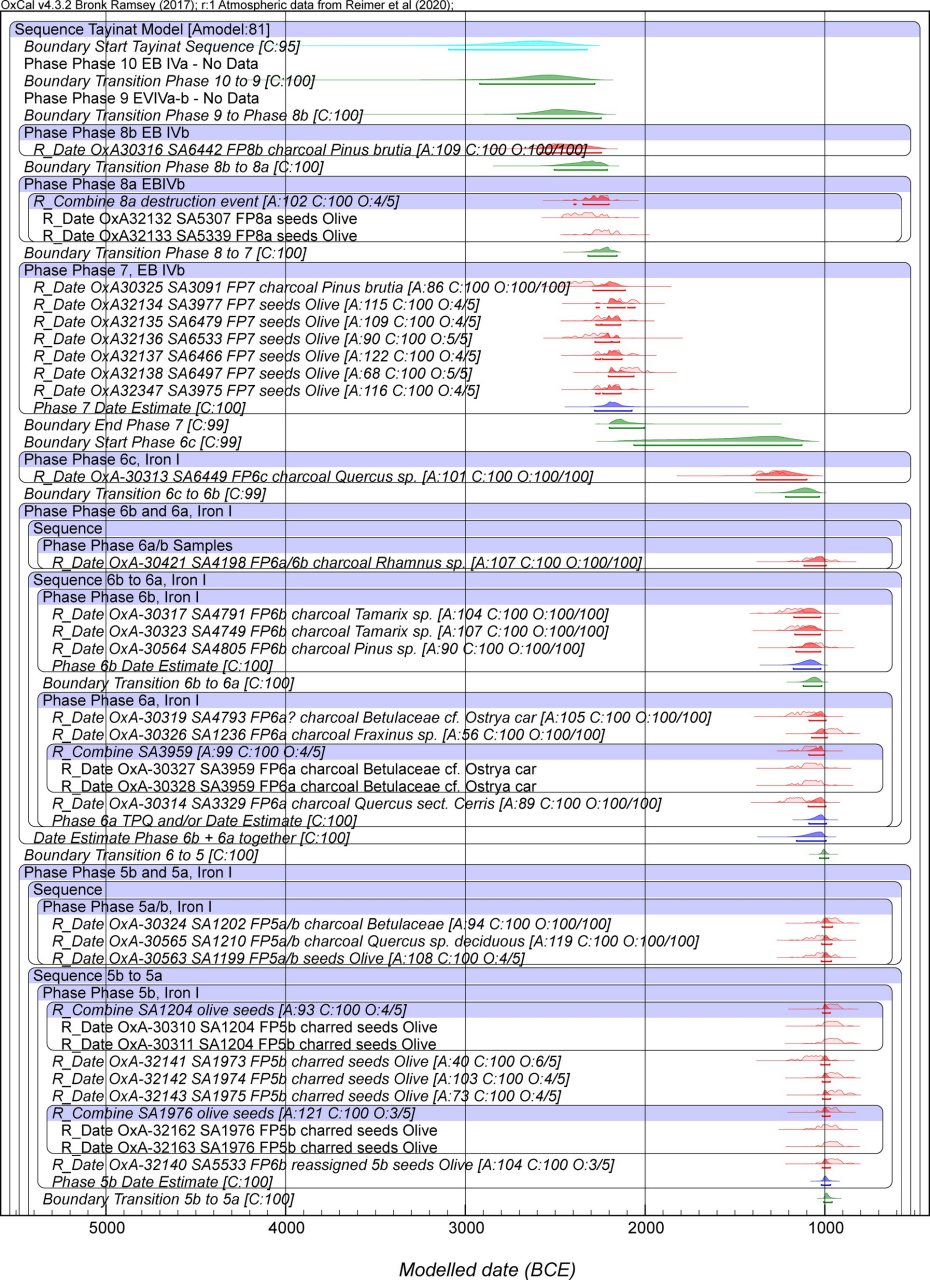
Model 2 (excluding OxA-32139): Bayesian chronological model for Tell Tayinat Iron Age sequence, part 1. The A_model_ and A_overall_ values are satisfactory versus the threshold value of 60 (Table 4). Data from OxCal 4.3.2 and IntCal20 with calibration curve resolution set at 1 year. The Individual OxCal Agreement values (A), the Posterior v. Prior values from the OxCal General Outlier model for the short-lived samples (O), and the Convergence (C) values are all shown. The wood charcoal samples with the Charcoal Plus Outlier model applied all have a Posterior/Prior value of 100/100. The light-shaded red probability distributions for each dated sample are the non-modeled calibrated age probability distributions for each sample in isolation. The dark red probability distributions are the modeled calendar age probability distributions. The line under each probability distribution indicates the modeled 95.4% highest posterior density (hpd) range. Cyan color indicates the start and end Boundaries of the model. Green color indicates the Boundaries calculated within the Tell Tayinat Sequence. Blue color indicates an OxCal Date estimate query for a Phase (this quantifies the time period within the start and end Boundary for the relevant Phase).

**Fig 4:**
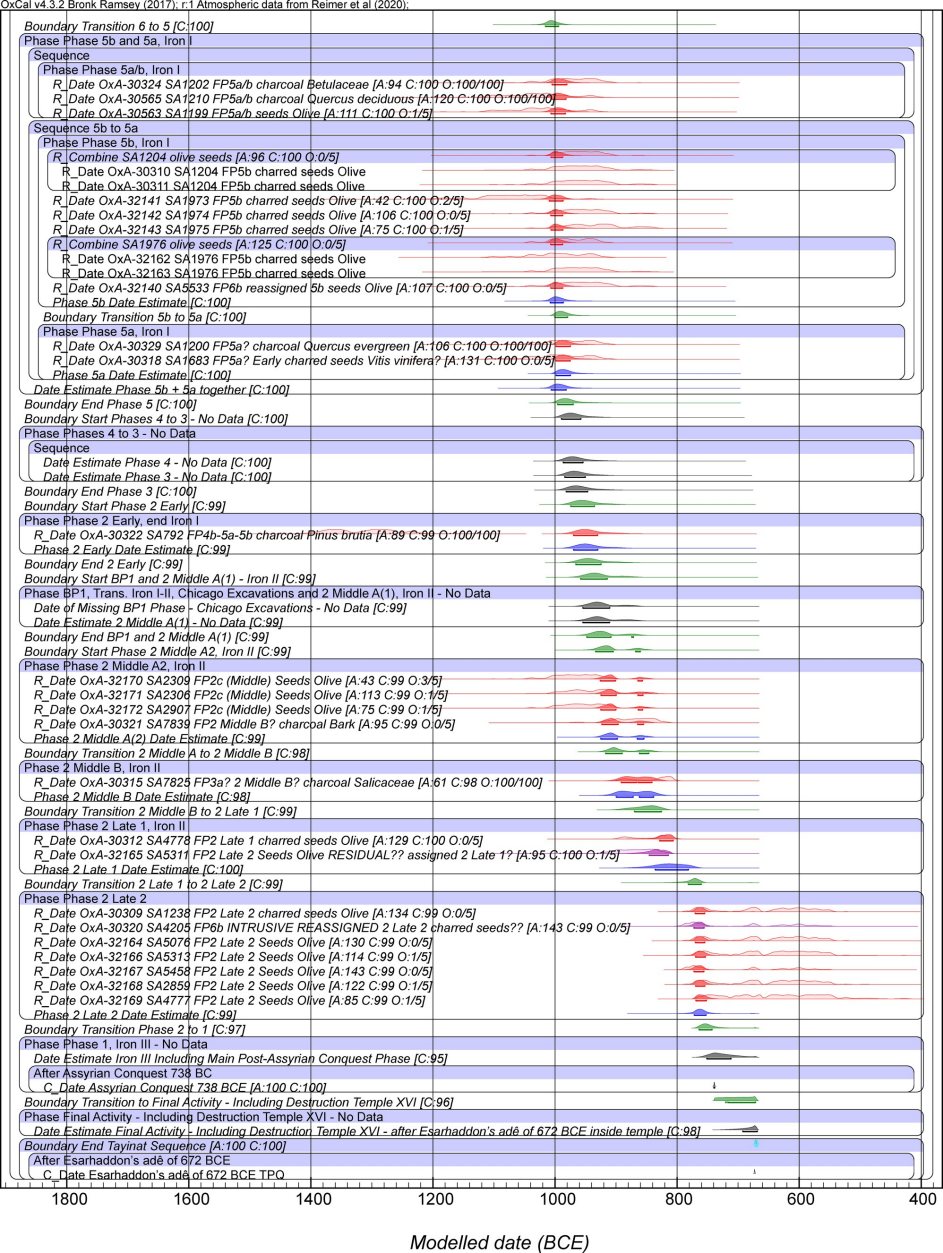
Model 2 (excluding OxA-32139): Bayesian chronological model for Tell Tayinat Iron Age sequence, part 2. Otherwise, see caption to Fig 3.

Figs 3 and 4 show Model 2. This is a revision of Model 1 removing OxA-32139. Model 2 achieves acceptable OxCal agreement index values overall, with A_model_ around 81 and A_overall_ around 81, above the accepted threshold value of 60 (typical values based on several runs). Usually all elements now achieve satisfactory Convergence values ≥95. There are no outliers among the dates on short-lived samples above the 6% level. These are very minor discrepancies, and the outlier modeling slightly down-weights their influence. We thus employ this model as our best estimate for the Tell Tayinat chronology. The calendar age ranges for various of the elements from both Model 1 and Model 2 are detailed in Table 4. They are very similar.

## Discussion

**Fig 5:**
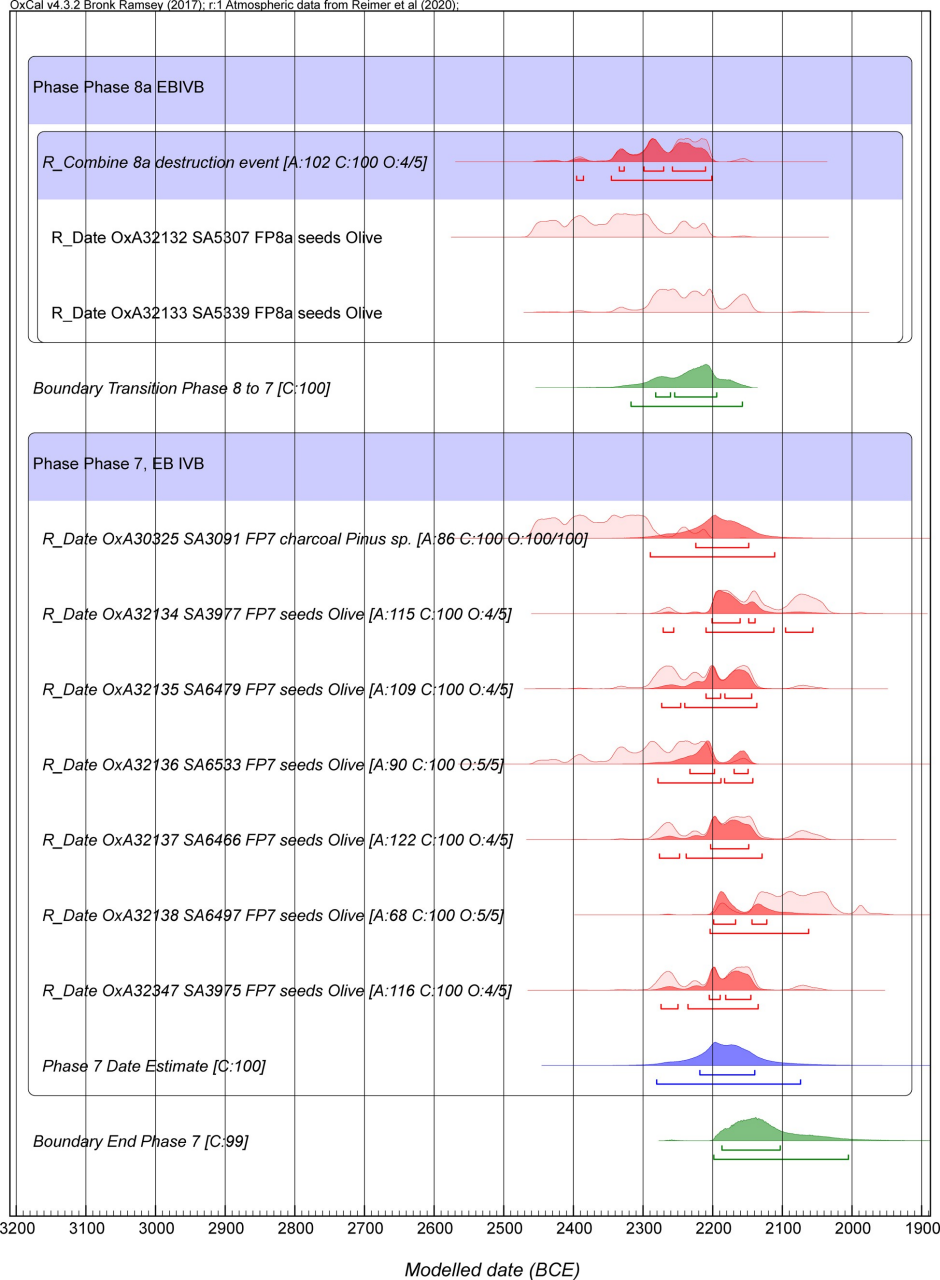
The Tell Tayinat Phase 7 data from Model 2 in Fig 3 shown in more detail. The lines under each probability distribution indicate the modeled 68.2% and 95.4% highest posterior density (hpd) ranges.

The EB IV dates indicate that the terminal EB occupation at Tell Tayinat lies in the 23^rd^ to 22^nd^ centuries BCE. This suggests that the site was active as the 4.2 ka climate event began (23^rd^ century BCE) and likely continued at least into the early part of the following century. We have relatively few data as yet from the more substantial earlier phases of occupation, including the construction of a major structure in FP8b [48, 50], for which only a *terminus post quem* can be provided. The destruction of this structure, however, appears to be dated somewhere between 2300–2200 BCE, relatively early in the EB IVB period. Furthermore, no dates have yet been obtained from the recently excavated preceding levels, most notably from another substantial construction that appears to have been destroyed by fire and has been tentatively dated to the EB IVA period (FP10). More data, however, pertains to the terminal phases of EB occupation, after the destruction of the FP8 structure. The final EB IV Phase 7, in particular, exhibits some spread in ^14^C ages (Figs 3 and 5) among the olive pits represented, with two (OxA-32138, OxA-32134) perhaps indicating a date range around/after 2100 BCE. Phase 7 appears to represent a relatively drawn out period of reducing circumstances at the site following the destruction of the more substantial architecture of Phase 8. We lack any constraint on the end of Phase 7, since there is then a gap in site occupation. The end Boundary could reach, in round terms, to ~2100 BCE at 68.2% hpd and ~2000 BCE at 95.4% hpd. The data to hand suggest that Tell Tayinat Phase 7 occupation likely ran at least well into the 22^nd^ century BCE, and perhaps further.

This phase is associated with the decline and eventual abandonment of the site at the end of the phase, representing the terminal EB occupation at the site, after which occupation shifts to the neighboring site of Tell Atchana (ancient Alalakh) for the Middle and Late Bronze Ages, beginning in the terminal phases of the EB. This abandonment thus appears to occupy a period around and shortly after the onset of the 4.2 ka cultural-climate episode. It has been increasingly recognized that this climate episode did not significantly impact sites in the northern Levant, and specifically sites in the Euphrates and Orontes river valleys, in the same manner as observed in northern Mesopotamia [34, 38]. This does not contradict the general climate shift to more arid conditions observed in a number of records, including directly from barley finds from archaeological sites in the region [144]. But it does highlight that such a general regional impact has very different effects locally, depending on a range of human, geographic/environmental and technological factors [144]. Even during the climate episodes around 4200 BP (~2200 BCE) and 3200 BP (~1200 BCE), barley grains from coastal sites, for example, show little substantive drought stress [144]. In the northern Levant, in particular, it has been proposed that the karstic geology of these regions and their resulting access to underground aquifers as more stable water sources in times of drought, positioned them as “refugia” for “habitat-tracking populations” in times of climate crisis [25, 145, see also ref. 35]. Favorable physiographic and edaphic features, or irrigation technology and low-risk, sustainable agricultural practices may also mitigate local impacts.

Such comparatively favorable conditions, as well as possible mitigation strategies, may be reflected in the archaeobotanical evidence from Tayinat. In both FP8 and FP7, barley and free-threshing wheat are well-represented, and emmer wheat is also present in significant quantities [48, 146, 147]. Although barley is the dominant cereal crop, the frequencies of (more water-demanding) wheat are notably higher compared to sites further east, which typically relied much more heavily on barley [148]. This suggests better water availability in the Amuq compared to other inland areas in the late 3^rd^ millennium, but the frequency of emmer may simultaneously represent a strategy aimed at minimizing yield variation. Similarly, Tayinat displays very high ubiquities in FPs 8–7 for a range of water-demanding species such as olive and grape, particularly when compared to other inland sites [146, 147]. Notably, no significant shift in species representation appears between FP8 and FP7 [147]. Carbon isotope analysis of cereal grains in addition indicates no evidence of drought stress in either barley or wheat during either phase, and crops may in fact be slightly better watered in FP7 compared to FP8 [147].

On the other hand, the zooarchaeological assemblages associated with FP8 and FP7 show notable differences [48]. Sheep and goat have a greater focus in absolute numbers compared to pig and cattle in FP7 in comparison to FP8, although cattle appear to have contributed the most significant amount of meat to the diet in both phases. Kill-off patterns for sheep and goat suggest a mixed animal management strategy was employed in both phases, with meat, milk and wool all likely playing a significant role. Notably, hunting is frequent in FP8, but becomes much less common in FP7. Hunting in FP8 focused on red deer (*Cervus elaphus*), although roe deer (*Capreolus capreolus*), gazelle and hare/rabbit are also present. Similarly, fish is found in very high proportions in FP8, and although it remains high in FP7 it declines significantly. Similar patterns are seen in bird, turtle and amphibian remains, although these are all found in much lower numbers than fish.

The results suggest a much more varied subsistence strategy in FP8, relying on a combination of domestic livestock and significant supplementation from hunting, fishing and fowling. In FP7, in contrast, hunting and fishing decrease notably and reliance on livestock increases. This is particularly true of sheep, goat and cattle, although pig declines slightly in FP7. Despite the decline visible architecturally after the destruction of the major building at the end of FP8, these observations suggest that FP7’s subsistence economy became more intensively focused on domestic livestock than in the preceding period.

Overall, despite their approximate temporal coincidence, a direct causal relationship between Tayinat’s late EB decline and this climate episode is difficult to support, as it seems much more likely that the site failed to recover from its destruction during the preceding century (or more–since the length of Phase 7 is unclear). This destruction is positioned alongside evidence for similar destruction levels in the northern Levant, most notably at the major regional center of Tell Mardikh-Ebla, where the Palace G complex was destroyed ~2350–2300 BCE [83]. The agents responsible for such destructions remain debated [149–151], but in the aftermath, Ebla was rapidly reconstructed and regained its status as a primary regional center, although perhaps on a more modest scale than observed in the preceding period [34, 39, 152]. Tayinat, in contrast, although re-occupied, does not seem to have recovered its primate position in the region, with the principal settlement shifting to nearby Tell Atchana, which then remained the central settlement throughout the Middle and Late Bronze Ages. This shift, in contrast to wider regional reconstructions of major climatic crisis, has often been postulated as the result of local factors, such as a decisive shift in the course of the Orontes River [153, 154]. Indeed, in light of the close proximity of Tell Tayinat and Tell Atchana, this change may be more reflective of a local political and spatial reorganization than a major break in settlement.

Our Iron Age data and modeling provide a refined and robust absolute timeframe for the early Iron Age at Tell Tayinat, independent of cultural and historical assumptions, running from around the 12^th^ through mid-8^th^ centuries BCE (Table 4, Figs 3 and 4). The site’s earliest Iron Age occupation thus lies squarely in the period following the collapse of the Late Bronze Age ca. 1200 BCE, and represents an alternative model and context for this period of transformation and climate challenge in the Old World [51]. Discussion here will focus primarily on the dates from the early Iron Age (i.e. Iron I-early Iron II, ca. 12^th^-9^th^ centuries BCE, Table 4); further discussion of the late Iron II dates here will await a future publication about the Iron II-III transition at the site (i.e. 8^th^-7^th^ centuries BCE) [155]. The dates calculated are consistent with the broad chronological sequence constructed based on linkages to Aegeanizing ceramics of the Late Helladic (LH) IIIC tradition [45, 46, 51, 55, 56]. The earliest levels (Field Phase [FP] 6c), which largely precede the widespread use of Aegeanizing LH IIIC-style ceramics at the site [51, 110], begin in the 12^th^ century BCE. Aegeanizing influences begin to appear in the late 12^th^ century (FP6b) and proliferate in the 11^th^ century (FP6a-5), continuing in declining frequencies into the mid-10^th^ century (FP4-3, 2 Early) [51, 56, 110]. These dates are consistent with a more or less conventional chronology and do not support recent suggestions for much earlier (or ‘higher’) dates for the end of the Late Bronze Age (and the LH IIIB to LH IIIC transition) [97]. We place Tell Tayinat Phase 6b-a and its assemblage and associations with the Late Helladic IIIC tradition [51, 56, 110] from the late 12^th^ century BCE onwards, in line with other recent ^14^C based work in the Aegean and East Mediterranean [94, 96, 156–158].

The original excavations at the site produced a monumental structure known as Building XIV that was assigned to Building Period 1 [53]. The current excavations have identified the foundations of this structure [45–47, 118], but have not yet produced any datable contexts for radiocarbon analysis. The stratigraphic position of the foundational remains of Building XIV, however, allow the proposition of a narrow date range, based on the model presented here, in the mid-late 10^th^ century BCE, contemporary with Phase 2 Middle A1. The latter phase is associated in Field 1 with the construction of a major ditch and sherd-paved street, located immediately to the south of Building XIV [118]. The material assemblage associated with this building and with the street contains Red-Slipped Burnished Ware, whose fluorescence is conventionally dated to the Iron Age II, although only small quantities were found [118]. Instead, the ceramic assemblage for phases 2 Middle A(1) and 2 Middle A(2) is dominated by plain wares, suggesting a date between Phases 2 Middle A and 2 Middle B (ca. 900 BCE) for the Iron I-II transition. Samples analyzed that post-date the Iron I-II transition at the site all originate from the Iron II occupation at Tell Tayinat, ending in Phase 2 Late 2. This appears likely to precede closely the 738 BCE date when the Assyrians conquered the site and assumed its control [65, 66, 118]. The modelled dates for Phase 2 Late (Table 4) are consistent with this assessment. The Date estimate is 772–753 BCE (68.2% hpd) and 791–735 BCE (95.4% hpd), indicating the period lies shortly before the Assyrian conquest. The Boundary estimate for the Phase 2 to 1 transition, 764–743 BCE (68.2% hpd), 771–721 BCE (95.4% hpd) is either just before, or at about the same time as, the Assyrian conquest. We currently lack any ^14^C data from Phase 1 to clarify the dating after Phase 2. It seems inherently likely that the Phase 2 to 1 transition probably is associated with the Assyrian conquest and associated changes following 738 BCE and this assumption appears compatible with the available ^14^C-based timeframe.

No Iron III context has yet been the subject of ^14^C dating, but the dates might thus be anticipated to fall in the period of Assyrian control following 738 BCE and must continue until after at least 672 BCE [66, 118, 143].

Our chronology allows comparison of some persons known from the epigraphic record and likely linked with names thought to refer to Tell Tayinat or its territory [47, 60–62] (see Table 5). The Aleppo Citadel inscription of king Taita, hero and ruler of Palistin, is dated to the 11^th^ century BCE on the basis of paleography and iconography [60–62]. The currently proposed chronological scheme would link this ruler to Tayinat Phases 6a to 5. A putative second Taita, reconstructed from inscriptional evidence from Meharde and Sheizar, has been attributed on similar grounds to the (early?) 10^th^ century BCE [62]. This ruler would thus most likely be associated with the later Iron I materials at the site (FPs 4–3). As such, contrary to earlier assumptions, the reigns of these rulers likely preceded the monumental constructions of Building Period 1 (Buildings XIII and XIV). This time period is designated Phase 2 Middle A(1). Dates are estimated within the model from the surrounding data and constraints, since there are no ^14^C data for this period, at about 955–911 BCE, 68.2% hpd, and 961–866 BCE, 95.4% hpd (Table 4). Any potential monumental constructions associated with the reigns of the earlier 10^th^ century rulers remain to be discovered. Other possible 10^th^ century rulers attested on the Arsuz stelae [62, 98], notably Suppiluliuma (I), who claims to have conducted a war in Cilicia, may be more plausibly related to the construction of the monumental structures associated with Building Period 1 in the mid-late 10^th^ century BCE. These prominent figures also appear to be associated with a significant reconfiguration of urban space at the site, and an expansion of their kingdom’s territorial extent. By the 9^th^ century BCE, Tell Tayinat had become the city of Kunulua, the apparent royal city of the Neo-Hittite kingdom of Patina (or Unqi) [47, 61, 159]. The ruler Suppiluliuma (II), attested in a monumental inscription recently discovered at Tell Tayinat, likely corresponds to the Sapalulme mentioned in the campaign records of Shalmaneser III [62, 63, 159, 160]. Qalparunda, a ruler of Patina, paid tribute to Shalmaneser III in both 857 and 853 BCE, and may correspond to the Halparuntiyas mentioned in Tayinat Inscription 1 [47, 159–161]. These two rulers would likely correspond with the time of Phase 2 Middle B.

**Table 5:** Suggested correlations between Tayinat Phases, absolute dates as reconstructed here by Bayesian modelling of ^14^C dates, rulers attested in historical sources, and conventional northern Levantine Iron Age periodization. For Iron I, alternating pale orange and white coloration denotes the four-period division as outlined in [51]; for Iron II-III, gray and white coloration denotes the separation between Tayinat Phases.

Absolute Dates (BCE)	Tayinat Phases	Historically Attested Kings, after [62]	General Iron Age Periodization, modified after [105, 106]
***Early 12***^***th***^ ***century***	6c		Iron IA
***Mid-12***^***th***^ ***century***			
***Late 12***^***th***^ ***century***	6b		
***Early 11***^***th***^ ***century***	6a		Iron IB
***Mid-11***^***th***^ ***century***		Taita I	
***Late 11***^***th***^ ***century***	5b		
	5a		
***Early 10***^***th***^ ***century***	4	Taita II	Iron IC
	3		
***Mid-10***^***th***^ ***century***	2 Early	Manana	
***Late 10***^***th***^ ***century***	2 Middle A(1), BP1	Suppiluliuma I	Iron I-II Transition
	2 Middle A(2)	Halparuntiya I	
***Early 9***^***th***^ ***century***	2 Middle B	Lubarna I?	
***Mid-9***^***th***^ ***century***		Suppiluliuma II (Sapalulme)	Iron IIA
		Qalparunda II	
***Late 9***^***th***^ ***century***	2 Late 1	Lubarna II, Surri/Sasi	
***Early 8***^***th***^ ***century***	2 Late 2		Iron IIB
***Mid-8***^***th***^ ***century***			
***Late 8***^***th***^ ***century***	1 (not dated)	Assyrian Conquest (738 BCE)	Iron III

Here too, a direct association between various reconstructions of 13^th^-10^th^ centuries BCE climatic crisis in the eastern Mediterranean and Near East (e.g. [3, 20–23, 26, 43, 162–164]) and the trajectory of development at the site of Tell Tayinat is difficult to discern. The decline of the Late Bronze Age settlement of Tell Atchana begins at least a century earlier than the proposed major crisis that occurs ca. 1200 BCE and afterwards, and the site’s occupation shrinks significantly during the 13^th^ century BCE [165, 166]. In contrast, during the proposed Early Iron Age shift to more arid conditions, the site of Tell Tayinat appears to flourish [51, 110]. Historical records suggest the formation and expansion of a major political entity centered around the Amuq Plain and Tell Tayinat during the 11^th^-10^th^ centuries BCE [60–62], the height of the reconstructed climate crisis. Although monumental architecture contemporary to the earliest historically attested rulers has not yet been uncovered by the current excavations, the archaeological evidence suggests a prosperous settlement that remained tied into long-distance trade networks. Major monumental constructions and a significant reorganization of urban space are attested in contexts now dated to the late 10^th^ century (Building Period 1, Phase 2 Middle A(1)), culminating in the formation of the major Neo-Hittite royal city of Kunulua in the 9^th^ century BCE. In combination with the historical evidence, this suggests that the rise of Tayinat, and the foundations of the later Iron Age social and political institutions at the site, had their origins during a period frequently defined (elsewhere in the wider region) as one of crisis and collapse.

The archaeological evidence from Tayinat points toward a variety of mechanisms that may have contributed to this Iron Age fluorescence. The stability of the location of the central settlement in the Amuq in the south-central part of the plain throughout the Bronze and Iron Ages suggests that the short-distance shift in site location from Tell Atchana to Tayinat during the 12^th^ century BCE could not have been a response to large-scale climatic degradation or to major shifts in economic networks. Rather, it seems likely to represent an adaptive response to locally changing conditions, which may have included a shift in the course of the Orontes River [153–154]. An Orontes paleo-channel identified between the sites of Tayinat and Atchana has been suggested to date to the Iron Age, and the shift of the main settlement to the north bank of the river would have maximized the site’s access to agricultural land in the plain north of the river [154]. Furthermore, the site of Tayinat al-Saghir, a small mound artificially constructed between Tayinat and Atchana at some point during the Iron Age, may represent a quay that would have allowed the main settlement to control riverine traffic, cementing its economically strategic position [118].

Palaeobotanical evidence likewise provides additional detail to this complex picture. Crop isotope data from Tayinat suggest minimal evidence for drought stress during the early Iron Age, particularly for barley, and generally suggest improvement in crop water status over the course of the Iron I [147, 167]. Furthermore, continued cultivation of water-demanding species such as grape, olive and fig at Tayinat during the Iron I does not suggest that water availability was a significant issue [167]. These arboricultural taxa reach their highest ubiquities and frequencies in FP6 and generally decline somewhat thereafter [51]. Free-threshing wheat also remains the most frequent crop plant throughout the Iron I, although barley increases in ubiquity during FPs 6–5 [51]. This increased focus on free-threshing wheat has been suggested to represent a labor optimization strategy [167]. Emmer is notably more ubiquitous during the Iron I than in the preceding LBA or during the later parts of the Iron Age, which may represent a strategy employed to minimize the risk associated with fluctuations in yield [51]. The frequency of large-seeded vetches such as bitter vetch (*Vicia*) and grass pea (*Lathyrus*) are similarly interpreted as indicative of a risk-spreading strategy that prevents crop failures, stabilizes crop yields and maintains soil fertility [167]. In the Iron Age, the frequent co-occurrence in northern Levantine sites of a focus on free-threshing wheat and large vetches has been interpreted as an attempt to achieve balance between the water input and labor requirements of agricultural production [167].

The zooarchaeological evidence points to a reasonably consistent strategy of animal raising through the Iron I, although differences exist between FP6b-a and the later phases of the Iron I. Pig consumption is at its most frequent during FP6b-a, although cattle consistently represents the most significant contributor of meat to the diet [51]. Mortality curves for sheep and goat in the Iron I suggest strategies aimed at exploitation of secondary products (dairy and particularly wool/hair), and there is evidence for significant textile production beginning already in the late 12^th^ century [51, 110]. Hunting evidence is found in very low frequencies in all phases, while fishing is noticeably more frequent in FP6b-a compared to later Iron I phases [51].

In addition to the political decline of Alalakh during the 13^th^ century BCE, textual sources suggest that problems with grain production and/or distribution may have resulted in the diversion of local agricultural products to elsewhere within the Hittite Empire [15, 42, 168], as well as a concurrent decline in regional population within the Amuq as a result of Hittite deportations that preceded the climate crisis [168]. The frequencies of wild taxa associated with moist environments suggest a wetter environment at Tayinat during FP6 [51, 147]. The Amuq has historically experienced a fluctuating degree of marshiness [118, 153], and this may explain the increased appearance of fish in the zooarchaeological assemblage during the same period [51]. In contrast, FPs 5–3 are associated with wild plant taxa that may indicate an expansion in agricultural area during the late 11^th^-10^th^ centuries BCE through the use of new arable fields that were likely left uncultivated during the 12^th^-early 11^th^ centuries BCE [51]. This suggests an agricultural extensification strategy during this time that may be related to increasing population in the Amuq Plain (and stable political-economic circumstances), as identified from settlement data [51, 56, 169], and which may reflect increasing urbanization connected to the formation of the kingdom of Palistin. Indeed, comparison of the apparent lower town plans extant and accessible via geophysics at Tell Tayinat would suggest that Tayinat records long-term organic town growth through the course of the Iron Age, whereas the nearby site of Zincirli, in contrast, perhaps largely records a new post-Assyrian conquest layout [169]. This may indicate only limited post-Assyrian-conquest changes beyond the monumental constructions of the elite zone in the upper city at Tayinat [118].

As suggested for the late EB period (see above), the local environment (through a combination of the Orontes River and the karstic geology) may have provided a suitable context in this region for greater resilience despite the arguments and evidence for climate change (more arid conditions) following ~3200 BP (~1200 BCE). Crop isotope data from Tayinat mentioned above are consistent with broader regional patterns that suggest the Orontes and coastal regions were somewhat less affected by drought stress [144, 167]. Comparative studies would also indicate that the nature of the association between climate change and social change is, predictably, complex and contingent on the nature and history of the relevant societies and their specific vulnerabilities (e.g. evolved socio-environmental mis-matches [170]). In particular, climate change alone is often not the key element that overcomes societal resilience; rather, it is sharp, volatile, fluctuations with a duration of several years that seem the greater threat to established complex agrarian societies in the preindustrial period (e.g. [29, 171–176]).

A final issue is the robustness of the ^14^C calibration record with respect to the Tell Tayinat samples. The new IntCal20 [133] ^14^C dataset is greatly enhanced (in terms of data for several periods, and quality) compared with IntCal13 [134]. However, for the periods of time relevant to Tell Tayinat and its dated elements, there are in fact only relatively small changes (see S3 Fig) and no major new underlying data contributions. Table 6 compares the results for Model 2 (in Table 4) with the same model run with IntCal13 (an example is cited with good OxCal A_model_ and A_overall_ values and all Convergence, C, values ≥95 –note that many runs do not achieve good Convergence in the final part of the model unless the kIterations value is increased, from about or after Phase 2 Late 1). There are few substantial differences; date ranges are largely similar.

**Table 6:** Comparison of the modeled calendar age ranges from Model 2 with IntCal20 [133] (Table 4) versus runs of Model 2 using the previous IntCal13 calibration curve [134] and the Hd GOR Mediterranean dataset [180]. Data from example runs with satisfactory A_model_ and A_overall_ values (>60) and with all dated elements with satisfactory Convergence values (≥95). Whole ranges listed. Phase 4 and 3 Date estimates combined as start Phase 4 to end Phase 3.

	Model 2 IntCal20,		Model 2 IntCal13		Model 2 Hd GOR	
	A_model_ 81, A_overall_ 81		A_model_ 71, A_overall_ 73		A_model_ 88, A_overall_ 88	
	*68*.*2% hpd*	*95*.*4% hpd*	*68*.*2% hpd*	*95*.*4% hpd*	*68*.*2% hpd*	*95*.*4% hpd*
	*Date BCE*	*Date BCE*	*Date BCE*	*Date BCE*	*Date BCE*	*Date BCE*
**Phase 8b EB IVB TPQ**	2517–2331	2585–2245	2518–2328	2587–2251	2519–2330	2588–2250
**Phase 8a, EBIVB Destruction Event**	2335–2211	2396–2202	2332–2215	2388–2203	2332–2215	2389–2203
**Phase 7 Date Estimate**	2219–2140	2281–2074	2223–2141	2280–2074	2223–2141	2281–2074
**Boundary End Phase 7**	2187–2104	2199–2006	2187–2101	2199–2012	2187–2101	2199–2009
**Phase 6c, Iron I TPQ**	1309–1159	1379–1101	1304–1158	1377–1101	1308–1138	1400–1084
**Phase 6b Date Estimate**	1122–1045	1176–1023	1121–1047	1173–1023	1105–1025	1162–1003
**Phase 6a TPQ and/or Date**	1052–1006	1089–992	1051–1005	1085–992	1037–994	1078–981
**Phase 5b Date Estimate**	1008–987	1019–970	1005–986	1017–973	996–976	1007–953
**Phase 5a Date Estimate**	998–976	1006–952	995–976	1004–958	986–955	991–940
**Time Span Phases 4&3 –No Samples**	987–951	997–920	985–954	995–932	971–928	981–905
**Phase 2 Early Date Estimate**	971–931	982–894	968–936	981–908	945–899	963–878
**Phase BP1, Chicago, and Phase 2 Middle A1 –No Data**	955–911	961–866	949–917	963–877	909–864	938–853
**Phase 2 Middle A2 Date Estimate**	926–855	933–841	927–897	934–850	889–843	926–835
**Phase 2 Middle B Date Estimate**	900–839	910–828	902–854	913–833	855–827	886–820
**Phase 2 Late 1 Date Estimate**	836–782	868–766	843–789	871–770	828–781	848–763
**Phase 2 Late 2 Date Estimate**	772–753	791–735	776–752	797–735	769–750	788–731
**Boundary Transition Phase 2 to 1**	764–743	771–721	764–742	767–721	759–739	766–717
**Assyrian Conquest**	738	738	738	738	738	738
**Boundary End Tayinat Sequence**	672–669	674–668	672–669	674–668	672–669	674–668

A potentially greater concern is recent work indicating the possible relevance at times of a modest/small Mediterranean growing season-related ^14^C offset for high-resolution calendar age determinations from the lower elevation Mediterranean region [177–180]. There is an intra-annual (i.e. seasonal) atmospheric ^14^C cycle, with a winter low and a summer high. Thus plants growing (and photosynthesizing) in the Mediterranean region at lower elevations in the winter through spring period (and stopping growth by the summer) may yield a recognizably slightly different ^14^C history versus the IntCal record derived from trees from central and northern Europe and North America that grow primarily from later spring and right through the summer [179, 180]. However, the fact that the overwhelming majority of the short-lived samples in this Tayinat study are olive pits (~86% of such samples) likely partly mitigates this issue. Unlike many trees or cereals and other field crops in lower elevation eastern Mediterranean environments, olive fruit grow from later spring through the autumn and hence comprise a ^14^C record that is only partly (not largely) out of kilter with the IntCal record. Grapes also grow through, and are harvested late in the summer to start of autumn, and again, in contrast with field crops like cereals, thus minimize any likely growing season offset [179–181] (in all, olive pits and a grape seed include 89% of the short-lived samples dated at Tayinat). Nonetheless, for interest, we compare the Model 2 results run against the Mediterranean-Anatolian Hd Gordion (GOR) dataset [180] in Table 6. This Hd GOR record is only a sketch for the Mediterranean—much more work is needed—and lacks data for the EB part of the Tayinat record and ends during the period of the late Tayinat samples. Thus it offers only a partial indication of possible differences. The Hd GOR record is largely similar to IntCal20 for most periods, and some of the changes in IntCal20, versus IntCal13, reduce what were previously further instances of differences when comparing the Hd GOR record versus IntCal13 [180], for example especially in the earlier 16^th^ century BCE (see the region labelled 1 in S4 Fig). Nonetheless, within general similarity, there are some periods, notably at the times of reversals and plateaus in the radiocarbon record, where the Hd GOR record exhibits some offset. Two examples are indicated (labelled as 2, 3) in S4 Fig. A possible additional area of minor offset might also exist at the reversal/plateau covering the earlier to mid-9^th^ century BCE labelled with the? in S4 Fig. The last two of these offsets could have minor relevance and effect on the Tayinat dates—but, noting that the types of short-lived samples dated likely minimize any effect (see above), this is likely insignificant.

As with the IntCal13 model, a number of model runs fail to achieve satisfactory Convergence values especially for the last part of the model from the Phase 2 Late 1 Date Estimate onwards unless the kIterations value is increased. Over multiple runs, there is also more noise in the late part of the model. The results in Table 6 are for a typical successful run with good Convergence. The results are generally similar to those from IntCal20 and IntCal13. However, in line with the observations of a small growing season offset issue and its possible consequences [177–180], we notice some modest effects, and especially during periods of reversals and plateaus in the radiocarbon calibration curve [179, 180]. For the periods where the Hd GOR [180] dataset applies (thus only for Tayinat Phases 6 onwards), the date ranges for some of the Tayinat phases (from Phase 6c to Phase 2 Middle B) are a little later, variously by around a decade to several decades considering the 68.2% hpd ranges. Consistent with previous observations [179, 180], the largest shift indications (of ~12–47 years in the 68.2% hpd ranges) occur in the 10^th^ and early to mid-9^th^ centuries BCE when there are reversals in the ^14^C calibration curve [133, 134] (S4 Fig). In view of the comments above about olive fruit and grapes, the effective (i.e. real) offset for Tell Tayinat is likely a little smaller. Nevertheless, this exercise highlights an area of possible minor chronological variation. Where this offset does apply, the effect is to achieve slightly later (more recent) calendar age estimates (something of potential relevance to debates over Iron Age chronology in the southern Levant, for example [179]). The very substantial change in atmospheric radiocarbon levels (the steep slope in the calibration curve) from the late 9^th^ through mid-8^th^ centuries BCE (linked with a major change in radiocarbon production and thence changes in solar activity processes [182]) (S4 Fig) clarifies that, regardless of any minor variations, the Tayinat Phase 2 Late 2 data are mid-8^th^ century BCE and thus likely represent the last pre-Assyrian conquest (738 BCE) phase at the site.

## Conclusions

The absolute dating of the later Early Bronze Age and earlier Iron Age occupation periods at Tell Tayinat and associated northern Levantine sites has been the subject of debate and ambiguity for many years. The regional chronological frameworks for the northern Levant during both periods have never been adequately addressed in absolute chronological terms, but rather have been largely based on relative chronologies derived from regional ceramic sequences. The chronology of the early Iron Age, in particular, has been linked only approximately to material and stylistic associations and thence to debates in other areas, in both the Aegean and the wider East Mediterranean, concerning the centuries following the collapse of the 13^th^ century BCE palace-era Late Bronze Age civilizations. Our chronology, based on the integration of the archaeological sequence at Tell Tayinat with radiocarbon dates, provides for the first time a directly relevant, refined, and robust timeframe for this important site and its region. The chronological framework thus developed places the two major occupation phases at Tell Tayinat firmly within temporal contexts relevant to ongoing debates about two periods of supposed climate crisis (around and following ca. 4200 BP/2200 BCE and around and following ca. 3200 BP/ 1200 BCE). The complex and contrasting responses observed at Tell Tayinat during these two transformative periods positions the site as a locus strategic to understanding the diverse ‘alternative’ developmental trajectories observed during these two intermediate eras.

## Supporting information

S1 File OxCal Runfiles for Model 1 and Model 2 and the.prior File for the Charcoal Plus Outlier model. (PDF)

S2 File Comparison of a portion of Model 2 (from Phases 6c through 5a) run without the application of the Charcoal Plus Outlier model (A) versus a run with the Charcoal Plus Outlier model applied (B) to illustrate the effect and importance of the Charcoal Plus Outlier model in order to achieve a likely and appropriate age model for Tell Tayinat integrating both data on long-lived charcoal samples (offering various TPQ ranges) and data on short-lived samples which (if in correct context association) offer contemporary age estimates.
(PDF)

S3 File Results for the selected elements of Model 2 as listed in Table 4 comparing the outcomes from a different model run (some results vary by typically around 1 year) with the Charcoal Plus Outlier model (as in the main text and Table 4) versus the same model run alternatively with the Charcoal Outlier model [135]. The Charcoal Plus Outlier version has just three elements with OxCal Agreement values <60 (e.g., typical example, OxA-30326 @56.5%, OxA-32141 @39.6%, OxA-32170 @41.4%), whereas the Charcoal Outlier version has four elements <60 (e.g., typical example, OxA-30326 @26.6%, OxA-32141 @36.1%, OxA-32170 @ 36.9%, and OxA-30315 @53.3%). The date ranges for the selected elements shown are nonetheless very similar. Whereas whole ranges are listed in Table 4, here sub-ranges are detailed where present.
(PDF)

S1 Fig Model 1: Bayesian chronological model for Tell Tayinat Iron Age sequence, part 1. Data from OxCal 4.3.2 [121, 132, 135] and IntCal20 [133] with calibration curve resolution set at 1 year. The Individual OxCal Agreement values (A), the Posterior v. Prior values from the OxCal General Outlier model for the short-lived samples (O), and Convergence values (C) are all shown. The wood charcoal samples with the Charcoal Plus Outlier model applied all have a Posterior/Prior value of 100/100. The light-shaded red probability distributions for each dated sample are the non-modeled calibrated age probability distributions for each sample in isolation. The dark red probability distributions are the modeled calendar age probability distributions. The lines under each probability distribution indicate the modeled 68.2% and 95.4% highest posterior density (hpd) ranges. Cyan color indicates the start and end Boundaries of the model. Green color indicates the Boundaries calculated within the Tell Tayinat Sequence. Blue color indicates an OxCal Date estimate for a Phase.
(TIF)

S2 Fig Model 1: Bayesian chronological model for Tell Tayinat Iron Age sequence, part 2. Otherwise, see captions to Fig 3, S1 Fig. The line under each probability distribution indicates the 95.4% hpd range.
(TIF)

S3 Fig Model 1 ^14^C dated elements (see S1 and S2 Figs) shown placed against the IntCal20 [133] calibration curve (and with the previous IntCal13 calibration curve [134] shown for comparison). (TIF)

S4 Fig The Heidelberg (Hd) Gordion (GOR) ^14^C dataset [180], 1σ, shown placed against IntCal20 [133] (to achieve satisfactory A_model_/A_overall_ values for a wiggle-match against IntCal20 after removing the 14 largest outliers in the dataset). The IntCal13 calibration curve [134] is shown for comparison. The labels indicate: 1. a region in the 16^th^ century BCE where previously there was an offset between the Hd GOR dataset and IntCal13 [180] but which is now largely removed with the revised IntCal20 dataset; 2. and 3. two regions (reversals and/or plateaus in the calibration curve) where there appear to be positive offsets between the Hd GOR data and IntCal20; and? another reversal and plateau where there is perhaps a small difference between the Hd GOR dataset and IntCal20. A. shows overall comparison, B. shows detail for the mid-12^th^ to 8^th^ centuries BCE.
(TIF)
