# Supplementary material for: Beyond megadrought and collapse in the Northern Levant: The chronology of Tell Tayinat and two historical inflection episodes, around 4.2ka BP, and following 3.2ka BP
Source: PLoS One. 2020 Oct 29;15(10):e0240799. doi: 10.1371/journal.pone.0240799 (PMC7595433; doi:10.1371/journal.pone.0240799)
Supplement: S1 File — (PDF) [file pone.0240799.s001.pdf]

OxCal runfiles for Model 1 and Model 2 and .prior File for Charcoal Plus Outlier Model

To be run with IntCal20 as the selected or default calibration curve. The .prior file (see below) is needed to run the Charcoal Plus Outlier model “IA” (for Inbuilt Age) as below. We regard this as the most appropriate option to allow for and to incorporate in-built age for the radiocarbon dates on long-lived charcoal samples in this analysis (following [136, 137]). The comparison of using the OxCal Charcoal Outlier model is discussed in File S3. We select a high kIterations value to reduce instances of runs completing with low Convergence values.

Model 1.

```
Options()
 {
  Resolution=1;
  kIterations=3000;
 };
 Plot()
 {
  Outlier_Model("IA",Prior("Charcoal_Plus"),U(0,3),"t");
  Outlier_Model("SL",T(5),U(0,4),"t");
  Outlier_Model("SSimple",N(0,2),0,"s");
  Sequence("Tayinat Model 1")
  {
   Boundary("Start Tayinat Sequence")
   {
    color="Red";
   };
   Phase ("Phase 10 EB IVA - No Data")
   {
   };
   Boundary ("Transition Phase 10 to 9")
   {
    color="Green";
   };
   Phase ("Phase 9 EVIVA-B - No Data")
   {
   };
   Boundary("Transition Phase 9 to Phase 8b")
   {
    color="Green";
   };
   Phase ("Phase 8b EB IVB")
   {
    R_Date("OxA30316 SA6442 FP8b charcoal Pinus brutia",4048,29)
    {
     Outlier("IA", 1);
    };
   };
   Boundary ("Transition Phase 8b to 8a")
   {
    color="Green";
   };
   Phase("Phase 8a EBIVB")
   {
    R_Combine ("8a destruction event")
```

```
 {
  Outlier("SL", 0.05);
  R_Date("OxA32132 SA5307 FP8a seeds Olive",3861,31)
  {
   Outlier("SSimple",0.05);
  };
  R_Date("OxA32133 SA5339 FP8a seeds Olive",3799,28)
  {
   Outlier("SSimple",0.05);
  };
 };
};
Boundary ("Transition Phase 8 to 7")
{
 color="Green";
};
Phase ("Phase 7, EB IVB")
{
 R_Date("OxA30325 SA3091 FP7 charcoal Pinus brutia",3871,29)
 {
  Outlier("IA", 1);
 };
 R_Date("OxA32134 SA3977 FP7 seeds Olive",3737,29)
 {
  Outlier("SL", 0.05);
 };
 R_Date("OxA32135 SA6479 FP7 seeds Olive",3784,30)
 {
  Outlier("SL", 0.05);
 };
 R_Date("OxA32136 SA6533 FP7 seeds Olive",3830,29)
 {
  Outlier("SL", 0.05);
 };
 R_Date("OxA32137 SA6466 FP7 seeds Olive",3765,30)
 {
  Outlier("SL", 0.05);
 };
 R_Date("OxA32138 SA6497 FP7 seeds Olive",3697,29)
 {
  Outlier("SL", 0.05);
 };
 R_Date("OxA32347 SA3975 FP7 seeds Olive",3772,26)
 {
  Outlier("SL", 0.05);
 };
 Date ("Phase 7 Date Estimate")
 {
  color="blue";
 };
};
Boundary ("End Phase 7")
{
 color="Green";
};
Interval("Duration Period Between Phase 7 & Phase 6 - Missing MB and LB");
Boundary ("Start Phase 6c")
```

```
   {
    color="Green";
   };
   Phase ("Phase 6c, Iron I")
   {
    R_Date("OxA-30313 SA6449 FP6c charcoal Quercus sp.",3038,28)
    {
     Outlier("IA", 1);
    };
   };
   Boundary ("Transition 6c to 6b")
   {
    color="Green";
   };
   Phase ("Phase 6b and 6a, Iron I")
   {
    Sequence()
    {
     Phase ("Phase 6a/b Samples")
     {
      R_Date("OxA-30421 SA4198 FP6a/6b charcoal Rhamnus sp.",2872,31)
      {
       Outlier("IA", 1);
      };
     };
    };
    Sequence ("6b to 6a, Iron I")
    {
     Phase ("Phase 6b, Iron I")
     {
      R_Date("OxA-30317 SA4791 FP6b charcoal Tamarix sp.",2962,27)
      {
       Outlier("IA", 1);
      };
      R_Date("OxA-30323 SA4749 FP6b charcoal Tamarix sp.",2929,28)
      {
       Outlier("IA", 1);
      };
      R_Date("OxA-30564 SA4805 FP6b charcoal Pinus brutia",2882,26)
      {
       Outlier("IA", 1);
      };
      //R_Date("OxA-30320 SA4205 FP6b INTRUSIVE charred seeds?? Reassigned 2 
Late 2",2546,27)
      //{
      // Outlier("SL", 0.05);
      // color="purple";
      //};
      R_Date("OxA-32139 SA5113 FP6b seeds Olive RESIDUAL OR PRE-
6c??",3717,30)
      {
       Outlier("SL", 0.05);
       color="orange";
      };
      //R_Date("OxA-32140 SA5533 FP6b seeds Olive",2806,30)
      //{
      // Outlier("SL", 0.05);
```

```
      //};
      //Reassigned to FP5b - see text discussion
      Date ("Phase 6b Date Estimate")
      {
       color="blue";
      };
     };
     Boundary ("Transition 6b to 6a")
     {
      color="Green";
     };
     Phase ("Phase 6a, Iron I")
     {
      R_Date("OxA-30319 SA4793 FP6a? charcoal Betulaceae cf. Ostrya
car",2918,27)
      {
       Outlier("IA", 1);
      };
      R_Date("OxA-30326 SA1236 FP6a charcoal Fraxinus sp.",2808,29)
      {
       Outlier("IA", 1);
      };
      R_Combine ("SA3959")
      {
       Outlier("SL", 0.05);
       R_Date("OxA-30327 SA3959 FP6a charcoal Betulaceae cf. Ostrya
car",2896,26)
       {
        Outlier("SSimple",0.05);
       };
       R_Date("OxA-30328 SA3959 FP6a charcoal Betulaceae cf. Ostrya
car",2891,27)
       {
        Outlier("SSimple",0.05);
       };
      };
      R_Date("OxA-30314 SA3329 FP6a charcoal Quercus sect. Cerris",2948,26)
      {
       Outlier("IA", 1);
      };
      Date ("Phase 6a TPQ and/or Date Estimate")
      {
       color="blue";
      };
     };
    };
    Date ("Date Estimate Phase 6b + 6a together")
    {
     color="blue";
    };
    Interval ("Duration Phase 6b + 6a");
   };
   Boundary ("Transition 6 to 5")
   {
    color="Green";
   };
   Phase ("Phase 5b and 5a, Iron I")
```

```
    {
     Sequence ()
     {
      Phase ("Phase 5a/b, Iron I")
      {
       R_Date("OxA-30324 SA1202 FP5a/b charcoal Betulaceae",2821,26)
       {
        Outlier("IA", 1);
       };
       R_Date("OxA-30565 SA1210 FP5a/b charcoal Quercus sp.
deciduous",2861,27)
       {
        Outlier("IA", 1);
       };
       R_Date("OxA-30563 SA1199 FP5a/b seeds Olive",2857,27)
       {
        Outlier("SL", 0.05);
       };
      };
     };
     Sequence ("5b to 5a")
     {
      Phase ("Phase 5b, Iron I")
      {
       R_Combine ("SA1204 olive seeds")
       {
        Outlier("SL", 0.05);
        R_Date("OxA-30310 SA1204 FP5b charred seeds Olive",2810,28)
        {
         Outlier("SSimple",0.05);
        };
        R_Date("OxA-30311 SA1204 FP5b charred seeds Olive",2806,31)
        {
         Outlier("SSimple",0.05);
        };
       };
       R_Date("OxA-32141 SA1973 FP5b charred seeds Olive",2886,28)
       {
        Outlier("SL", 0.05);
       };
       R_Date("OxA-32142 SA1974 FP5b charred seeds Olive",2805,30)
       {
        Outlier("SL", 0.05);
       };
       R_Date("OxA-32143 SA1975 FP5b charred seeds Olive",2786,29)
       {
        Outlier("SL", 0.05);
       };
       R_Combine ("SA1976 olive seeds")
       {
        Outlier("SL", 0.05);
        R_Date("OxA-32162 SA1976 FP5b charred seeds Olive",2839,26)
        {
         Outlier("SSimple",0.05);
        };
        R_Date("OxA-32163 SA1976 FP5b charred seeds Olive",2811,27)
        {
```

```
        Outlier("SSimple",0.05);
       };
      };
      R_Date("OxA-32140 SA5533 FP6b reassigned 5b seeds Olive",2806,30)
      {
       Outlier("SL", 0.05);
      };
      //OxA-32140 reassigned from FP6b to FP5b
      Date ("Phase 5b Date Estimate")
      {
       color="blue";
      };
     };
     Boundary ("Transition 5b to 5a")
     {
      color="Green";
     };
     Phase ("Phase 5a, Iron I")
     {
      R_Date("OxA-30329 SA1200 FP5a? charcoal Quercus evergreen",2829,27)
      {
       Outlier("IA", 1);
      };
      R_Date("OxA-30318 SA1683 FP5a? Early charred seeds Vitis
vinifera?",2837,27)
      {
       Outlier("SL", 0.05);
      };
      Date("Phase 5a Date Estimate")
      {
       color="blue";
      };
     };
    };
    Date ("Date Estimate Phase 5b + 5a together")
    {
     color="blue";
    };
    Interval ("Duration Phase 5b + 5a");
   };
   Boundary ("End Phase 5")
   {
    color="Green";
   };
   Boundary("Start Phases 4 to 3 - No Data");
   Phase("Phases 4 to 3 - No Data")
   {
    Sequence ()
    {
     Date("Date Estimate Phase 4 - No Data");
     Date("Date Estimate Phase 3 - No Data");
    };
    Interval("Duration Phases 4 to 3 - No Data");
   };
   Boundary("End Phase 3");
   Boundary ("Start Phase 2 Early")
   {
```

```
  color="Green";
 };
 Phase ("Phase 2 Early, end Iron I")
 {
  R_Date("OxA-30322 SA792 FP4b-5a-5b charcoal Pinus brutia",3047,26)
  {
   Outlier("IA", 1);
  };
  Date ("Phase 2 Early Date Estimate")
  {
   color="blue";
  };
 };
 Boundary ("End 2 Early")
 {
  color="Green";
 };
 Boundary("Start BP1 and 2 Middle A(1) - Iron II")
 {
  color="Green";
 };
 Phase ("BP1, Trans. Iron I-II, Chicago Excavations and 2 Middle A(1), Iron
II - No Data")
 {
  Date ("Date of Missing BP1 Phase - Chicago Excavations - No Data");
  Date("Date Estimate 2 Middle A(1) - No Data");
 };
 Boundary("End BP1 and 2 Middle A(1)")
 {
  color="Green";
 };
 Boundary("Start Phase 2 Middle A2, Iron II")
 {
  color="Green";
 };
 Phase ("Phase 2 Middle A2, Iron II")
 {
  R_Date("OxA-32170 SA2309 FP2c (Middle) Seeds Olive",2814,26)
  {
   Outlier("SL", 0.05);
  };
  R_Date("OxA-32171 SA2306 FP2c (Middle) Seeds Olive",2784,27)
  {
   Outlier("SL", 0.05);
  };
  R_Date("OxA-32172 SA2907 FP2c (Middle) Seeds Olive",2798,27)
  {
   Outlier("SL", 0.05);
  };
  R_Date("OxA-30321 SA7839 FP2 Middle B? charcoal Bark",2739,26)
  {
   Outlier("SL", 0.05);
  };
  Date ("Phase 2 Middle A(2) Date Estimate")
  {
   color="blue";
  };
```

```
   };
   Boundary ("Transition 2 Middle A to 2 Middle B")
   {
    color="Green";
   };
   Phase ("2 Middle B, Iron II")
   {
    R_Date("OxA-30315 SA7825 FP3a? 2 Middle B? charcoal Salicaceae",2679,27)
    {
     Outlier("IA", 1);
    };
    Date ("Phase 2 Middle B Date Estimate")
    {
     color="blue";
    };
   };
   Boundary("Transition 2 Middle B to 2 Late 1")
   {
    color="Green";
   };
   Phase ("Phase 2 Late 1, Iron II")
   {
    R_Date("OxA-30312 SA4778 FP2 Late 1 charred seeds Olive",2679,28)
    {
     Outlier("SL", 0.05);
    };
    R_Date("OxA-32165 SA5311 FP2 Late 2 Seeds Olive RESIDUAL?? assigned 2
Late 1?",2732,27)
    {
     Outlier("SL", 0.05);
     color="purple";
    };
    Date ("Phase 2 Late 1 Date Estimate")
    {
     color="blue";
    };
   };
   Boundary ("Transition 2 Late 1 to 2 Late 2")
   {
    color="Green";
   };
   Phase ("Phase 2 Late 2")
   {
    R_Date("OxA-30309 SA1238 FP2 Late 2 charred seeds Olive",2519,26)
    {
     Outlier("SL", 0.05);
    };
    R_Date("OxA-30320 SA4205 FP6b INTRUSIVE REASSIGNED 2 Late 2 charred
seeds??",2546,27)
    {
     Outlier("SL", 0.05);
     color="purple";
    };
    R_Date("OxA-32164 SA5076 FP2 Late 2 Seeds Olive",2516,26)
    {
     Outlier("SL", 0.05);
    };
```

```
    R_Date("OxA-32166 SA5313 FP2 Late 2 Seeds Olive",2506,25)
    {
     Outlier("SL", 0.05);
    };
    R_Date("OxA-32167 SA5458 FP2 Late 2 Seeds Olive",2545,25)
    {
     Outlier("SL", 0.05);
    };
    R_Date("OxA-32168 SA2859 FP2 Late 2 Seeds Olive",2511,25)
    {
     Outlier("SL", 0.05);
    };
    R_Date("OxA-32169 SA4777 FP2 Late 2 Seeds Olive",2486,26)
    {
     Outlier("SL", 0.05);
    };
    Date ("Phase 2 Late 2 Date Estimate")
    {
     color="blue";
    };
   };
   Boundary("Transition Phase 2 to 1")
   {
    color="Green";
   };
   Phase ("Phase 1, Iron III - No Data")
   {
    Date("Date Estimate Iron III Including Main Post-Assyrian Conquest
Phase");
    After("Assyrian Conquest 738 BCE")
    {
     C_Date("Assyrian Conquest 738 BCE",-738,0);
    };
   };
   Boundary("Transition to Final Activity - Including Destruction Temple
XVI")
   {
    color="Green";
   };
   Phase("Final Activity - Including Destruction Temple XVI - No Data")
   {
    Date("Date Estimate Final Activity - Including Destruction Temple XVI -
after Esarhaddon’s adê of 672 BCE inside temple");
   };
   Boundary("End Tayinat Sequence",After("Esarhaddon’s adê of 672
BCE"){C_Date("Esarhaddon’s adê of 672 BCE TPQ",-672,0);};)
   {
    color="Red";
   };
  };
 };
```

Model 2.

```
Options()
 {
  Resolution=1;
  kIterations=3000;
 };
 Plot()
 {
  Outlier_Model("IA",Prior("Charcoal_Plus"),U(0,3),"t");
  Outlier_Model("SL",T(5),U(0,4),"t");
  Outlier_Model("SSimple",N(0,2),0,"s");
  Sequence("Tayinat Model 2")
  {
   Boundary("Start Tayinat Sequence")
   {
    color="Red";
   };
   Phase ("Phase 10 EB IVA - No Data")
   {
   };
   Boundary ("Transition Phase 10 to 9")
   {
    color="Green";
   };
   Phase ("Phase 9 EVIVA-B - No Data")
   {
   };
   Boundary("Transition Phase 9 to Phase 8b")
   {
    color="Green";
   };
   Phase ("Phase 8b EB IVB")
   {
    R_Date("OxA30316 SA6442 FP8b charcoal Pinus brutia",4048,29)
    {
     Outlier("IA", 1);
    };
   };
   Boundary ("Transition Phase 8b to 8a")
   {
    color="Green";
   };
   Phase("Phase 8a EBIVB")
   {
    R_Combine ("8a destruction event")
    {
     Outlier("SL", 0.05);
     R_Date("OxA32132 SA5307 FP8a seeds Olive",3861,31)
     {
      Outlier("SSimple",0.05);
     };
     R_Date("OxA32133 SA5339 FP8a seeds Olive",3799,28)
     {
      Outlier("SSimple",0.05);
     };
    };
```

```
};
Boundary ("Transition Phase 8 to 7")
{
 color="Green";
};
Phase ("Phase 7, EB IVB")
{
 R_Date("OxA30325 SA3091 FP7 charcoal Pinus brutia",3871,29)
 {
  Outlier("IA", 1);
 };
 R_Date("OxA32134 SA3977 FP7 seeds Olive",3737,29)
 {
  Outlier("SL", 0.05);
 };
 R_Date("OxA32135 SA6479 FP7 seeds Olive",3784,30)
 {
  Outlier("SL", 0.05);
 };
 R_Date("OxA32136 SA6533 FP7 seeds Olive",3830,29)
 {
  Outlier("SL", 0.05);
 };
 R_Date("OxA32137 SA6466 FP7 seeds Olive",3765,30)
 {
  Outlier("SL", 0.05);
 };
 R_Date("OxA32138 SA6497 FP7 seeds Olive",3697,29)
 {
  Outlier("SL", 0.05);
 };
 R_Date("OxA32347 SA3975 FP7 seeds Olive",3772,26)
 {
  Outlier("SL", 0.05);
 };
 Date ("Phase 7 Date Estimate")
 {
  color="blue";
 };
};
Boundary ("End Phase 7")
{
 color="Green";
};
Interval("Duration Period Between Phase 7 & Phase 6 - Missing MB and LB");
Boundary ("Start Phase 6c")
{
 color="Green";
};
Phase ("Phase 6c, Iron I")
{
 R_Date("OxA-30313 SA6449 FP6c charcoal Quercus sp.",3038,28)
 {
  Outlier("IA", 1);
 };
};
Boundary ("Transition 6c to 6b")
```

```
   {
    color="Green";
   };
   Phase ("Phase 6b and 6a, Iron I")
   {
    Sequence()
    {
     Phase ("Phase 6a/b Samples")
     {
      R_Date("OxA-30421 SA4198 FP6a/6b charcoal Rhamnus sp.",2872,31)
      {
       Outlier("IA", 1);
      };
     };
    };
    Sequence ("6b to 6a, Iron I")
    {
     Phase ("Phase 6b, Iron I")
     {
      R_Date("OxA-30317 SA4791 FP6b charcoal Tamarix sp.",2962,27)
      {
       Outlier("IA", 1);
      };
      R_Date("OxA-30323 SA4749 FP6b charcoal Tamarix sp.",2929,28)
      {
       Outlier("IA", 1);
      };
      R_Date("OxA-30564 SA4805 FP6b charcoal Pinus brutia",2882,26)
      {
       Outlier("IA", 1);
      };
      //R_Date("OxA-30320 SA4205 FP6b INTRUSIVE charred seeds?? Reassigned 2
Late 2",2546,27)
      //{
      // Outlier("SL", 0.05);
      // color="purple";
      //};
      //R_Date("OxA-32139 SA5113 FP6b seeds Olive RESIDUAL OR PRE-
6c??",3717,30)
      //{
      // Outlier("SL", 0.05);
      //color="orange";
      //};
      //OxA-32139 excluded as massive outlier, residual sample, for Model 2
      //R_Date("OxA-32140 SA5533 FP6b seeds Olive",2806,30)
      //{
      // Outlier("SL", 0.05);
      //};
      //Reassigned to FP5b - see text discussion
      Date ("Phase 6b Date Estimate")
      {
       color="blue";
      };
     };
     Boundary ("Transition 6b to 6a")
     {
      color="Green";
```

```
     };
     Phase ("Phase 6a, Iron I")
     {
      R_Date("OxA-30319 SA4793 FP6a? charcoal Betulaceae cf. Ostrya
car",2918,27)
      {
       Outlier("IA", 1);
      };
      R_Date("OxA-30326 SA1236 FP6a charcoal Fraxinus sp.",2808,29)
      {
       Outlier("IA", 1);
      };
      R_Combine ("SA3959")
      {
       Outlier("SL", 0.05);
       R_Date("OxA-30327 SA3959 FP6a charcoal Betulaceae cf. Ostrya
car",2896,26)
        {
         Outlier("SSimple",0.05);
        };
       R_Date("OxA-30328 SA3959 FP6a charcoal Betulaceae cf. Ostrya
car",2891,27)
        {
         Outlier("SSimple",0.05);
        };
      };
      R_Date("OxA-30314 SA3329 FP6a charcoal Quercus sect. Cerris",2948,26)
      {
       Outlier("IA", 1);
      };
      Date ("Phase 6a TPQ and/or Date Estimate")
      {
       color="blue";
      };
     };
    };
    Date ("Date Estimate Phase 6b + 6a together")
    {
     color="blue";
    };
    Interval ("Duration Phase 6b + 6a");
   };
   Boundary ("Transition 6 to 5")
   {
    color="Green";
   };
   Phase ("Phase 5b and 5a, Iron I")
   {
    Sequence ()
    {
     Phase ("Phase 5a/b, Iron I")
     {
      R_Date("OxA-30324 SA1202 FP5a/b charcoal Betulaceae",2821,26)
      {
       Outlier("IA", 1);
      };
```

```
      R_Date("OxA-30565 SA1210 FP5a/b charcoal Quercus sp. 
deciduous",2861,27)
      {
       Outlier("IA", 1);
      };
      R_Date("OxA-30563 SA1199 FP5a/b seeds Olive",2857,27)
      {
       Outlier("SL", 0.05);
      };
     };
    };
    Sequence ("5b to 5a")
    {
     Phase ("Phase 5b, Iron I")
     {
      R_Combine ("SA1204 olive seeds")
      {
       Outlier("SL", 0.05);
       R_Date("OxA-30310 SA1204 FP5b charred seeds Olive",2810,28)
       {
        Outlier("SSimple",0.05);
       };
       R_Date("OxA-30311 SA1204 FP5b charred seeds Olive",2806,31)
       {
        Outlier("SSimple",0.05);
       };
      };
      R_Date("OxA-32141 SA1973 FP5b charred seeds Olive",2886,28)
      {
       Outlier("SL", 0.05);
      };
      R_Date("OxA-32142 SA1974 FP5b charred seeds Olive",2805,30)
      {
       Outlier("SL", 0.05);
      };
      R_Date("OxA-32143 SA1975 FP5b charred seeds Olive",2786,29)
      {
       Outlier("SL", 0.05);
      };
      R_Combine ("SA1976 olive seeds")
      {
       Outlier("SL", 0.05);
       R_Date("OxA-32162 SA1976 FP5b charred seeds Olive",2839,26)
       {
        Outlier("SSimple",0.05);
       };
       R_Date("OxA-32163 SA1976 FP5b charred seeds Olive",2811,27)
       {
        Outlier("SSimple",0.05);
       };
      };
      R_Date("OxA-32140 SA5533 FP6b reassigned 5b seeds Olive",2806,30)
      {
       Outlier("SL", 0.05);
      };
      //OxA-32140 reassigned from FP6b to FP5b
      Date ("Phase 5b Date Estimate")
```

```
      {
       color="blue";
      };
     };
     Boundary ("Transition 5b to 5a")
     {
      color="Green";
     };
     Phase ("Phase 5a, Iron I")
     {
      R_Date("OxA-30329 SA1200 FP5a? charcoal Quercus evergreen",2829,27)
      {
       Outlier("IA", 1);
      };
      R_Date("OxA-30318 SA1683 FP5a? Early charred seeds Vitis
vinifera?",2837,27)
      {
       Outlier("SL", 0.05);
      };
      Date("Phase 5a Date Estimate")
      {
       color="blue";
      };
     };
    };
    Date ("Date Estimate Phase 5b + 5a together")
    {
     color="blue";
    };
    Interval ("Duration Phase 5b + 5a");
   };
   Boundary ("End Phase 5")
   {
    color="Green";
   };
   Boundary("Start Phases 4 to 3 - No Data");
   Phase("Phases 4 to 3 - No Data")
   {
    Sequence ()
    {
     Date("Date Estimate Phase 4 - No Data");
     Date("Date Estimate Phase 3 - No Data");
    };
    Interval("Duration Phases 4 to 3 - No Data");
   };
   Boundary("End Phase 3");
   Boundary ("Start Phase 2 Early")
   {
    color="Green";
   };
   Phase ("Phase 2 Early, end Iron I")
   {
    R_Date("OxA-30322 SA792 FP4b-5a-5b charcoal Pinus brutia",3047,26)
    {
     Outlier("IA", 1);
    };
    Date ("Phase 2 Early Date Estimate")
```

```
 {
  color="blue";
 };
};
Boundary ("End 2 Early")
{
 color="Green";
};
Boundary("Start BP1 and 2 Middle A(1) - Iron II")
{
 color="Green";
};
Phase ("BP1, Trans. Iron I-II, Chicago Excavations and 2 Middle A(1), Iron
II - No Data")
{
 Date ("Date of Missing BP1 Phase - Chicago Excavations - No Data");
 Date("Date Estimate 2 Middle A(1) - No Data");
};
Boundary("End BP1 and 2 Middle A(1)")
{
 color="Green";
};
Boundary("Start Phase 2 Middle A2, Iron II")
{
 color="Green";
};
Phase ("Phase 2 Middle A2, Iron II")
{
 R_Date("OxA-32170 SA2309 FP2c (Middle) Seeds Olive",2814,26)
 {
  Outlier("SL", 0.05);
 };
 R_Date("OxA-32171 SA2306 FP2c (Middle) Seeds Olive",2784,27)
 {
  Outlier("SL", 0.05);
 };
 R_Date("OxA-32172 SA2907 FP2c (Middle) Seeds Olive",2798,27)
 {
  Outlier("SL", 0.05);
 };
 R_Date("OxA-30321 SA7839 FP2 Middle B? charcoal Bark",2739,26)
 {
  Outlier("SL", 0.05);
 };
 Date ("Phase 2 Middle A(2) Date Estimate")
 {
  color="blue";
 };
};
Boundary ("Transition 2 Middle A to 2 Middle B")
{
 color="Green";
};
Phase ("2 Middle B, Iron II")
{
 R_Date("OxA-30315 SA7825 FP3a? 2 Middle B? charcoal Salicaceae",2679,27)
 {
```

```
     Outlier("IA", 1);
    };
    Date ("Phase 2 Middle B Date Estimate")
    {
     color="blue";
    };
   };
   Boundary("Transition 2 Middle B to 2 Late 1")
   {
    color="Green";
   };
   Phase ("Phase 2 Late 1, Iron II")
   {
    R_Date("OxA-30312 SA4778 FP2 Late 1 charred seeds Olive",2679,28)
    {
     Outlier("SL", 0.05);
    };
    R_Date("OxA-32165 SA5311 FP2 Late 2 Seeds Olive RESIDUAL?? assigned 2
Late 1?",2732,27)
    {
     Outlier("SL", 0.05);
     color="purple";
    };
    Date ("Phase 2 Late 1 Date Estimate")
    {
     color="blue";
    };
   };
   Boundary ("Transition 2 Late 1 to 2 Late 2")
   {
    color="Green";
   };
   Phase ("Phase 2 Late 2")
   {
    R_Date("OxA-30309 SA1238 FP2 Late 2 charred seeds Olive",2519,26)
    {
     Outlier("SL", 0.05);
    };
    R_Date("OxA-30320 SA4205 FP6b INTRUSIVE REASSIGNED 2 Late 2 charred
seeds??",2546,27)
    {
     Outlier("SL", 0.05);
     color="purple";
    };
    R_Date("OxA-32164 SA5076 FP2 Late 2 Seeds Olive",2516,26)
    {
     Outlier("SL", 0.05);
    };
    R_Date("OxA-32166 SA5313 FP2 Late 2 Seeds Olive",2506,25)
    {
     Outlier("SL", 0.05);
    };
    R_Date("OxA-32167 SA5458 FP2 Late 2 Seeds Olive",2545,25)
    {
     Outlier("SL", 0.05);
    };
    R_Date("OxA-32168 SA2859 FP2 Late 2 Seeds Olive",2511,25)
```

```
    {
     Outlier("SL", 0.05);
    };
    R_Date("OxA-32169 SA4777 FP2 Late 2 Seeds Olive",2486,26)
    {
     Outlier("SL", 0.05);
    };
    Date ("Phase 2 Late 2 Date Estimate")
    {
     color="blue";
    };
   };
   Boundary("Transition Phase 2 to 1")
   {
    color="Green";
   };
   Phase ("Phase 1, Iron III - No Data")
   {
    Date("Date Estimate Iron III Including Main Post-Assyrian Conquest
Phase");
    After("Assyrian Conquest 738 BCE")
    {
     C_Date("Assyrian Conquest 738 BCE",-738,0);
    };
   };
   Boundary("Transition to Final Activity - Including Destruction Temple
XVI")
   {
    color="Green";
   };
   Phase("Final Activity - Including Destruction Temple XVI - No Data")
   {
    Date("Date Estimate Final Activity - Including Destruction Temple XVI -
after Esarhaddon’s adê of 672 BCE inside temple");
   };
   Boundary("End Tayinat Sequence",After("Esarhaddon’s adê of 672
BCE"){C_Date("Esarhaddon’s adê of 672 BCE TPQ",-672,0);};)
   {
    color="Red";
   };
  };
 };
```

Prior File for the Charcoal Plus Outlier Model

The data listing for a file "/IA.prior" for use with OxCal in order to run the Charcoal Plus Outlier model [136, 137] in an OxCal runfile with the line of code "`Outlier_Model("IA",Prior("Charcoal_Plus"),U(0,3),"t");`" (compare with the rounded data for the same file in Table S6 in ref. 136 electronic supplementary material available at: 2013http://rspa.royalsocietypublishing.org/content/royprsa/suppl/2013/09/02/rspa.2013.0395.DC1/rspa20130395supp1.pdf).

-10	4.31299E-05
-9.9	4.76659E-05
-9.8	5.2679E-05
-9.7	5.82193E-05
-9.6	6.43423E-05
-9.5	7.11092E-05
-9.4	7.85879E-05
-9.3	8.6853E-05
-9.2	9.59874E-05
-9.1	0.000106083
-9	0.000117239
-8.9	0.000129569
-8.8	0.000143196
-8.7	0.000158257
-8.6	0.000174901
-8.5	0.000193295
-8.4	0.000213624
-8.3	0.000236091
-8.2	0.000260921
-8.1	0.000288362
-8	0.000318689
-7.9	0.000352206
-7.8	0.000389248
-7.7	0.000430186
-7.6	0.000475429
-7.5	0.00052543
-7.4	0.00058069
-7.3	0.000641762
-7.2	0.000709257
-7.1	0.00078385
-7	0.000866288
-6.9	0.000957396
-6.8	0.001058086
-6.7	0.001169366
-6.6	0.00129235
-6.5	0.001428267

-6.4	0.001578479
-6.3	0.00174449
-6.2	0.001927959
-6.1	0.002130724
-6	0.002354815
-5.9	0.002602473
-5.8	0.002876177
-5.7	0.003178667
-5.6	0.003512971
-5.5	0.003882433
-5.4	0.004290752
-5.3	0.004742014
-5.2	0.005240736
-5.1	0.005791909
-5	0.00640105
-4.9	0.007074254
-4.8	0.00781826
-4.7	0.008640513
-4.6	0.009549244
-4.5	0.010553547
-4.4	0.011663473
-4.3	0.012890131
-4.2	0.014245798
-4.1	0.015744042
-4	0.017399857
-3.9	0.019229816
-3.8	0.021252233
-3.7	0.02348735
-3.6	0.025957536
-3.5	0.028687514
-3.4	0.031704606
-3.3	0.035039009
-3.2	0.038724094
-3.1	0.042796742
-3	0.047297715
-2.9	0.052272059
-2.8	0.057769559
-2.7	0.063845237
-2.6	0.070559899
-2.5	0.077980749
-2.4	0.086182056
-2.3	0.095245902
-2.2	0.105263
-2.1	0.116333607
-2	0.128568519
-1.9	0.142090188

-1.8	0.157033944
-1.7	0.173549348
-1.6	0.191801692
-1.5	0.211973652
-1.4	0.234267116
-1.3	0.258905203
-1.2	0.286134501
-1.1	0.31622753
-1	0.349485469
-0.9	0.386241177
-0.8	0.426862516
-0.7	0.471756039
-0.6	0.521371054
-0.5	0.576204127
-0.4	0.636804044
-0.3	0.70377731
-0.2	0.777794215
-0.1	0.859595547
0	0.95
0.1	0.045241871
0.2	0.040936538
0.3	0.037040911
0.4	0.033516002
0.5	0.030326533
0.6	0.027440582
0.7	0.024829265
0.8	0.022466448
0.9	0.020328483
1	0.018393972
1.1	0.016643554
1.2	0.015059711
1.3	0.01362659
1.4	0.012329848
1.5	0.011156508
1.6	0.010094826
1.7	0.009134176
1.8	0.008264944
1.9	0.007478431
2	0.006766764
2.1	0.006122821
2.2	0.005540158
2.3	0.005012942
2.4	0.004535898
2.5	0.00410425
2.6	0.003713679
2.7	0.003360276

2.8	0.003040503
2.9	0.002751161
3	0.002489353
3.1	0.00225246
3.2	0.00203811
3.3	0.001844158
3.4	0.001668663
3.5	0.001509869
3.6	0.001366186
3.7	0.001236176
3.8	0.001118539
3.9	0.001012096
4	0.000915782
4.1	0.000828634
4.2	0.000749779
4.3	0.000678428
4.4	0.000613867
4.5	0.00055545
4.6	0.000502592
4.7	0.000454764
4.8	0.000411487
4.9	0.000372329
5	0.000336897
5.1	0.000304837
5.2	0.000275828
5.3	0.00024958
5.4	0.000225829
5.5	0.000204339
5.6	0.000184893
5.7	0.000167298
5.8	0.000151378
5.9	0.000136972
6	0.000123938
6.1	0.000112143
6.2	0.000101472
6.3	9.18152E-05
6.4	8.30779E-05
6.5	7.5172E-05
6.6	6.80184E-05
6.7	6.15456E-05
6.8	5.56888E-05
6.9	5.03893E-05
7	4.55941E-05
7.1	4.12552E-05
7.2	3.73293E-05
7.3	3.37769E-05

7.4	3.05626E-05
7.5	2.76542E-05
7.6	2.50226E-05
7.7	2.26414E-05
7.8	2.04867E-05
7.9	1.85372E-05
8	1.67731E-05
8.1	1.5177E-05
8.2	1.37327E-05
8.3	1.24258E-05
8.4	1.12434E-05
8.5	1.01734E-05
8.6	9.20529E-06
8.7	8.32929E-06
8.8	7.53665E-06
8.9	6.81945E-06
9	6.17049E-06
9.1	5.58329E-06
9.2	5.05197E-06
9.3	4.57121E-06
9.4	4.1362E-06
9.5	3.74259E-06
9.6	3.38644E-06
9.7	3.06417E-06
9.8	2.77258E-06
9.9	2.50873E-06
10	2.27E-06
