# Supplementary material for: Beyond megadrought and collapse in the Northern Levant: The chronology of Tell Tayinat and two historical inflection episodes, around 4.2ka BP, and following 3.2ka BP
Source: PLoS One. 2020 Oct 29;15(10):e0240799. doi: 10.1371/journal.pone.0240799 (PMC7595433; doi:10.1371/journal.pone.0240799)
Supplement: S2 File — Comparison of a portion of Model 2 (from Phases 6c through 5a) run without the application of the Charcoal Plus Outlier model (A) versus a run with the Charcoal Plus Outlier model applied (B) to illustrate the effect and importance of the Charcoal Plus Outlier model in order to achieve a likely and appropriate age model for Tell Tayinat integrating both data on long-lived charcoal samples (offering various TPQ ranges) and data on short-lived samples which (if in correct context association) offer contemporary age estimates. (PDF) [file pone.0240799.s002.pdf]

## File S2

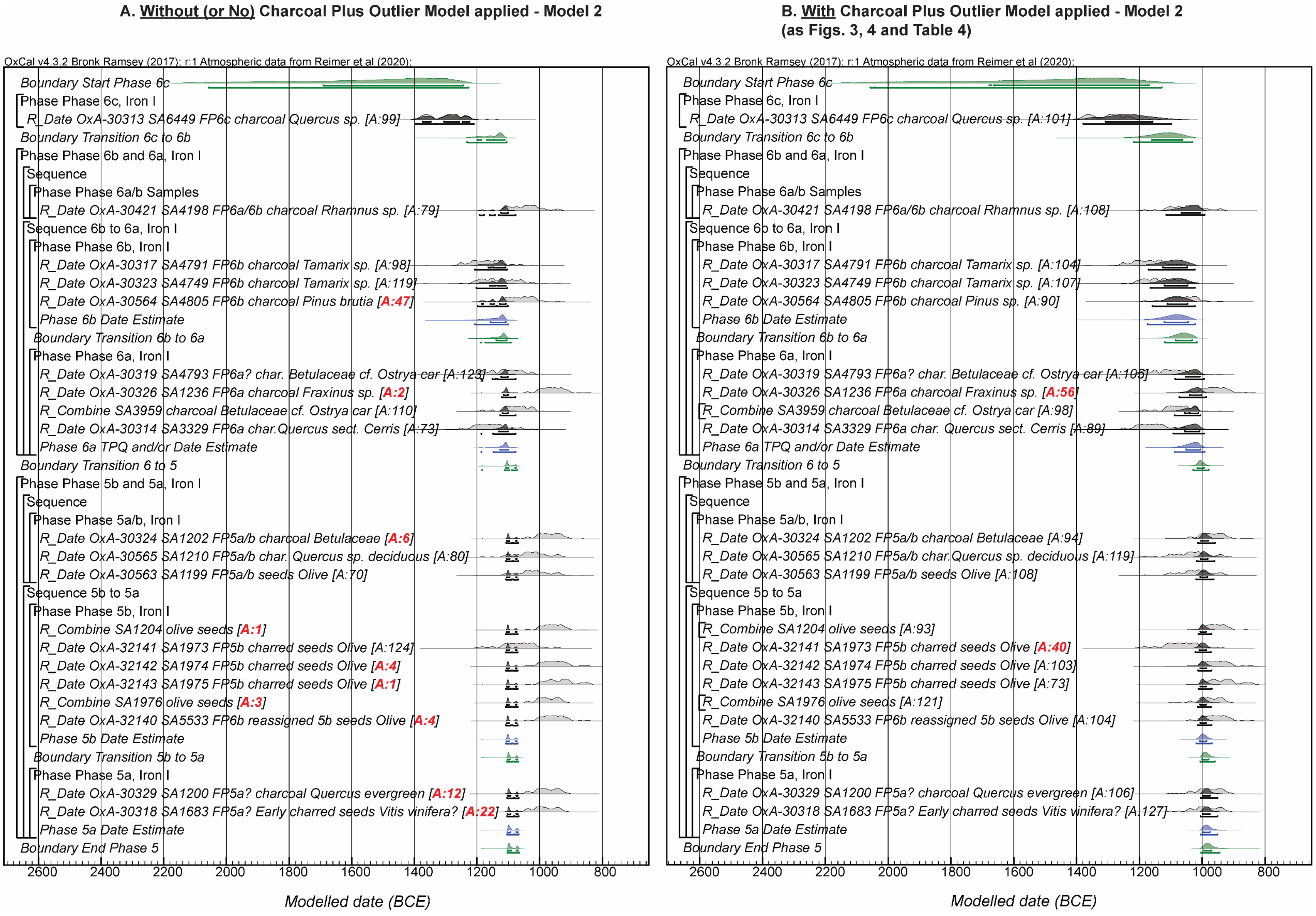


The File S2 examples shown above compare a portion of Model 2 (from Phases 6c through 5a) run without the application of the Charcoal Plus Outlier model (A) versus a run with the Charcoal Plus Outlier model applied (B) to illustrate the effect and importance

of the Charcoal Plus Outlier model in order to achieve a likely and appropriate age model for Tell Tayinat integrating both data on long-lived charcoal samples (offering various TPQ ranges) and data on short-lived samples which (if in correct context association) offer contemporary age estimates. Data using OxCal 4.3.2 [121, 132, 135] and IntCal20 [133] with curve resolution set at 1 year. The no Charcoal Plus Outlier model [136, 137] run (A) leaves 10 dates (or weighted averages) with poor individual agreement (OxCal A values <60), 9 of these as very poor (<15). The problem is the charcoal samples which in several cases include in-built age (or old wood effect) in Phase 6c (OxA-30313) and Phases 6b and 6a (OxA-30421, OxA-30317, OxA-30323, OxA-30564, OxA-30319, OxA-30326, R_Combine SA3959, OxA30314). With no modelling constraint to suggest that these are TPQ estimates, these dates force the group to place around 1100 BCE, and so leave the subsequent Phase 5b and 5a data pushed much too early – and hence all the poor agreement values since these dates (mainly on short-lived samples) clearly go later. In contrast, the model run with the Charcoal Plus Outlier model applied (B), avoids this problem. The various Phase 6b and 6a charcoal samples are now modelled towards the later part of the possible probability range for each of these dates and as more consistent with the more recent dates within the groupings (following the logic of the Charcoal outlier model [ref. 135] and tree allometry, where it is assumed that a set of charcoal TPQ ages form an exponential distribution, with a few dates much older than the context (innermost wood), some dates rather older than the context (inner to outer wood), and likely most dates from not that far before, to close to, the context from outer to outermost tree rings). The model with the Charcoal Plus Outlier model places Phase 5b and 5a around and following 1000 BCE consistent with the dates on the short-lived samples from these contexts. Only two dates now have poor individual OxCal agreement values, one only marginally so at A=56<60 (OxA-30316), and the other, OxA32141 (A=40<60), is just slightly too old an age for the context (small outlier or possible residual sample from Phase 6). A case like this is dealt with through the General Outlier model (down-weighting this older outlier in this case) in the Model 2 run and data shown in Figs. 3, 4 and Table 4.
