# Supplementary material for: Beyond megadrought and collapse in the Northern Levant: The chronology of Tell Tayinat and two historical inflection episodes, around 4.2ka BP, and following 3.2ka BP
Source: PLoS One. 2020 Oct 29;15(10):e0240799. doi: 10.1371/journal.pone.0240799 (PMC7595433; doi:10.1371/journal.pone.0240799)
Supplement: S3 File — The Charcoal Plus Outlier version has just three elements with OxCal Agreement values <60 (e.g., typical example, OxA-30326 @56.5%, OxA-32141 @39.6%, OxA-32170 @41.4%), whereas the Charcoal Outlier version has four elements <60 (e.g., typical example, OxA-30326 @26.6%, OxA-32141 @36.1%, OxA-32170 @ 36.9%, and OxA-30315 @53.3%). The date ranges for the selected elements shown are nonetheless very similar. Whereas whole ranges are listed in Table 4, here sub-ranges are detailed where present. (PDF) [file pone.0240799.s003.pdf]

File S3

Results for the selected elements of Model 2 as listed in Table 4 comparing the outcomes from a different model run (some results vary by typically around 1 year) with the Charcoal Plus Outlier model (as in the main text and Table 4) versus the same model run alternatively with the Charcoal Outlier model [135]. The Charcoal Plus Outlier version has just three elements with OxCal Agreement values <60 (e.g., typical example, OxA-30326 @56.5%, OxA-32141 @39.6%, OxA-32170 @41.4%), whereas the Charcoal Outlier version has four elements <60 (e.g., typical example, OxA-30326 @26.6%, OxA-32141 @36.1%, OxA-32170 @ 36.9%, and OxA-30315 @53.3%). The date ranges for the selected elements shown are nonetheless very similar. Whereas whole ranges are listed in Table 4, here sub-ranges are detailed where present.

	**Model 2 *excluding* OxA-32139 with Charcoal Plus Outlier model (as Table 4) A_{model} ~81, $A_{overall}$ ~80-81**		**Model 2 *excluding* OxA-32139 with Charcoal Outlier model A_{model} ~66, $A_{overall}$ ~66**	
	68.2% hpd Date BCE	***95.4% hpd Date BCE***	***68.2% hpd Date BCE***	***95.4% hpd Date BCE***
**Phase 8b EB IVB TPQ**	2518-2330	2585-2245	2514-2337	2581-2251
**Phase 8a, EBIVB Destruction Event**	2334-2328 (3.9)<br>2299-2271 (25.9)<br>2258-2211 (38.3)	2396-2386 (1.5)<br>2346-2202 (93.9)	2335-2327 (5.0)<br>2299-2271 (26.2)<br>2258-2211 (37.0)	2397-2387 (1.6)<br>2346-2202 (93.8)
**Phase 7 Date Estimate**	2219-2140	2281-2075	2219-2140	2282-2075
**Boundary End Phase 7**	2187-2104	2200-2007	2187-2105	2199-2006
**Phase 6c, Iron I TPQ**	1309-1159	1379-1100	1300-1155	1367-1102
**Phase 6b Date Estimate**	1122-1045	1175-1023	1110-1039	1164-1021
**Phase 6a TPQ and/or Date**	1052-1006	1088-992	1047-1004	1081-989
**Phase 5b Date Estimate**	1008-987	1019-969	1007-984	1018-964
**Phase 5a Date Estimate**	998-975	1006-951	997-972	1004-946
**Phase 4 Date estimate – No Samples**	987-955	997-925	986-951	995-920
**Phase 3 Date estimate – No Samples**	985-951	995-920	983-947	993-916
**Phase 2 Early Date Estimate**	971-930	982-893	970-926	980-890
**Phase BP1, Chicago, and Phase 2 Middle A1 – No Data**	954-911 (66.7)<br>883-881 (1.5)	961-866	951-913 (58.0)<br>889-877 (10.2)	959-864
**Phase 2 Middle A2 Date Estimate**	925-898 (54.6)<br>866-854 (13.6)	933-887 (66.0)<br>878-841 (29.4)	923-899 (45.1)<br>868-851 (23.1)	931-886 (58.1)<br>880-841 (37.3)
**Phase 2 Middle B Date Estimate**	900-872 (35.8)<br>863-839 (43.4)	910-827	888-871 (20.1)<br>868-837 (48.1)	906-827
**Phase 2 Late 1 Date Estimate**	836-782	868-766	834-784	859-767
**Phase 2 Late 2 Date Estimate**	772-753	793-733	773-753	794-733
**Boundary Transition Phase 2 to 1**	764-743	771-717	765-743	771-715 (94.9)<br>677-675 (0.5)
**Assyrian Conquest**	738	738	738	738
**Boundary End Tayinat Sequence**	672-669	674-668	672-669	674-668
