# Supplementary figures and images for: Beyond megadrought and collapse in the Northern Levant: The chronology of Tell Tayinat and two historical inflection episodes, around 4.2ka BP, and following 3.2ka BP
Source: PLoS One. 2020 Oct 29;15(10):e0240799. doi: 10.1371/journal.pone.0240799 (PMC7595433; doi:10.1371/journal.pone.0240799)

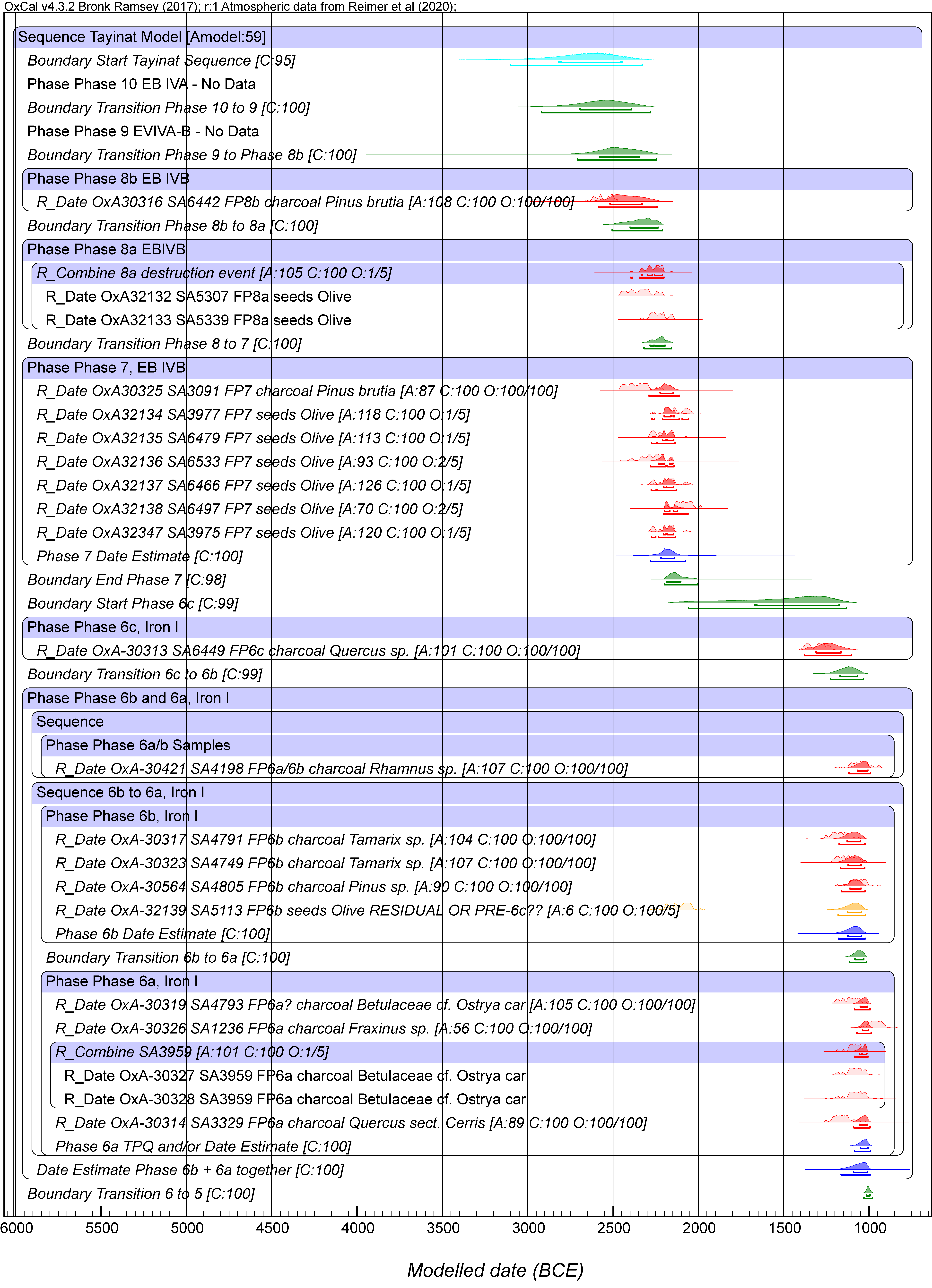

Supplement: S1 Fig — Data from OxCal 4.3.2 [121, 132, 135] and IntCal20 [133] with calibration curve resolution set at 1 year. The Individual OxCal Agreement values (A), the Posterior v. Prior values from the OxCal General Outlier model for the short-lived samples (O), and Convergence values (C) are all shown. The wood charcoal samples with the Charcoal Plus Outlier model applied all have a Posterior/Prior value of 100/100. The light-shaded red probability distributions for each dated sample are the non-modeled calibrated age probability distributions for each sample in isolation. The dark red probability distributions are the modeled calendar age probability distributions. The lines under each probability distribution indicate the modeled 68.2% and 95.4% highest posterior density (hpd) ranges. Cyan color indicates the start and end Boundaries of the model. Green color indicates the Boundaries calculated within the Tell Tayinat Sequence. Blue color indicates an OxCal Date estimate for a Phase. (TIF) [file pone.0240799.s004.tif]

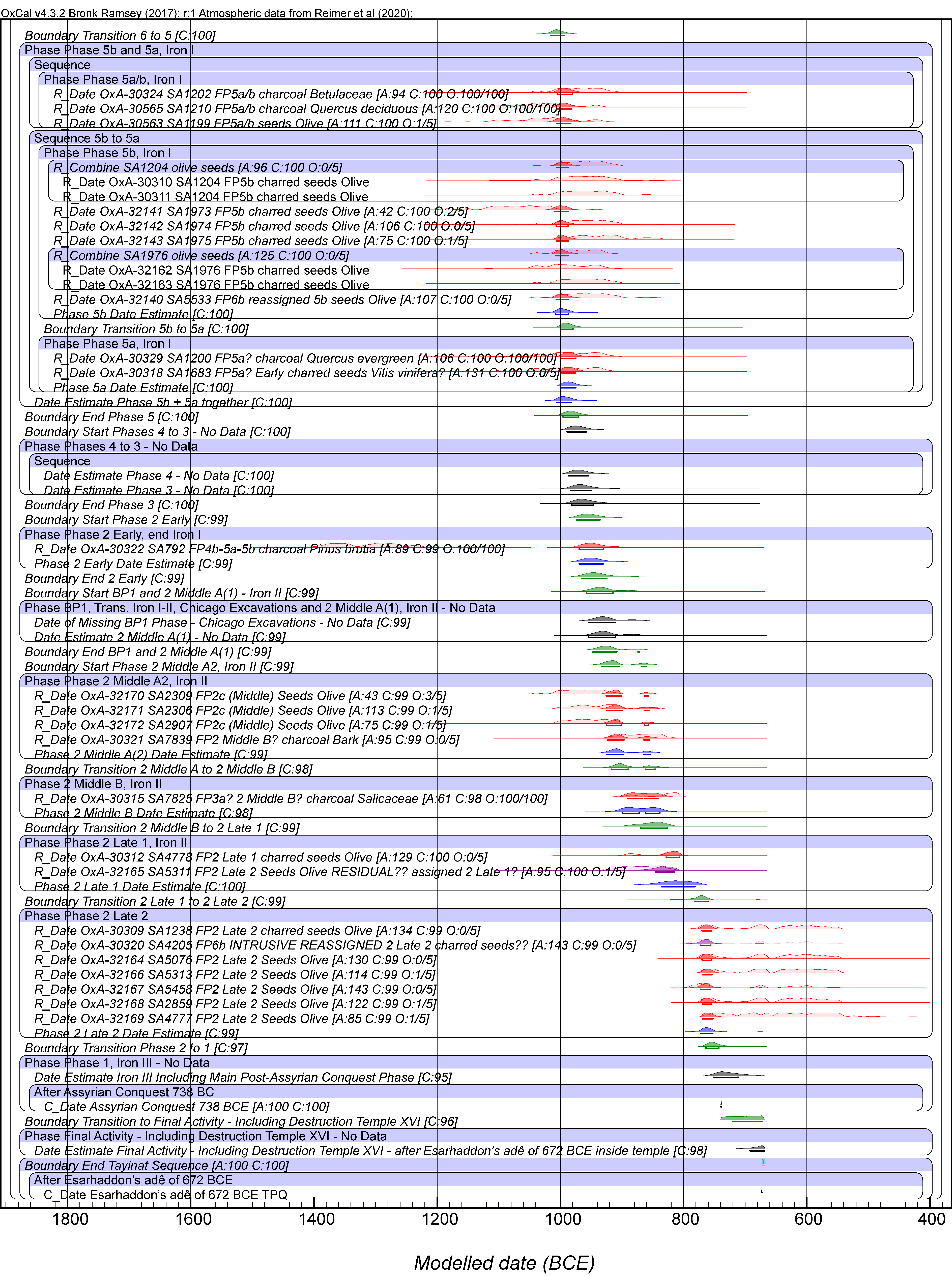

Supplement: S2 Fig — Otherwise, see captions to Fig 3, S1 Fig. The line under each probability distribution indicates the 95.4% hpd range. (TIF) [file pone.0240799.s005.tif]

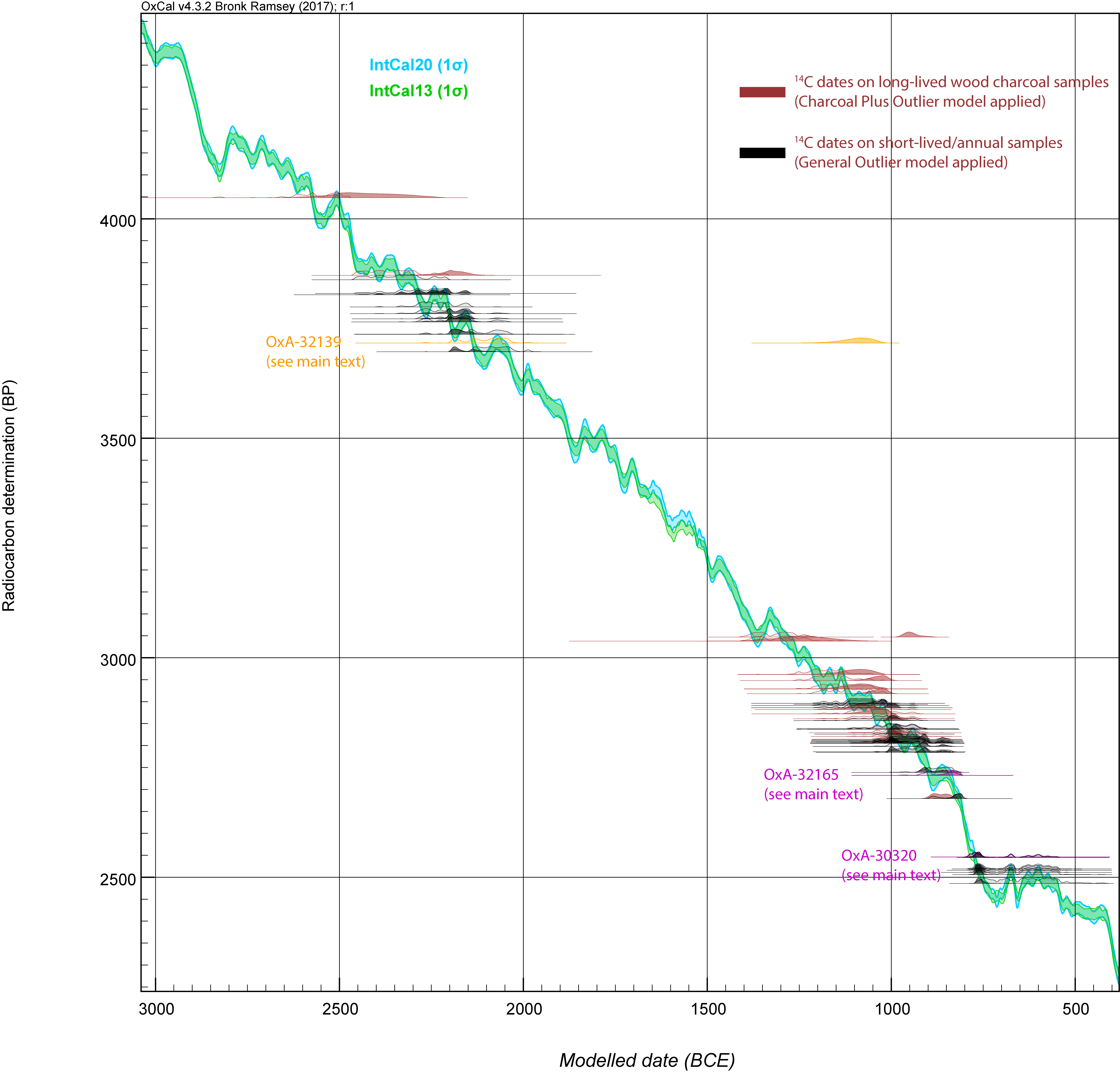

Supplement: S3 Fig — (TIF) [file pone.0240799.s006.tif]

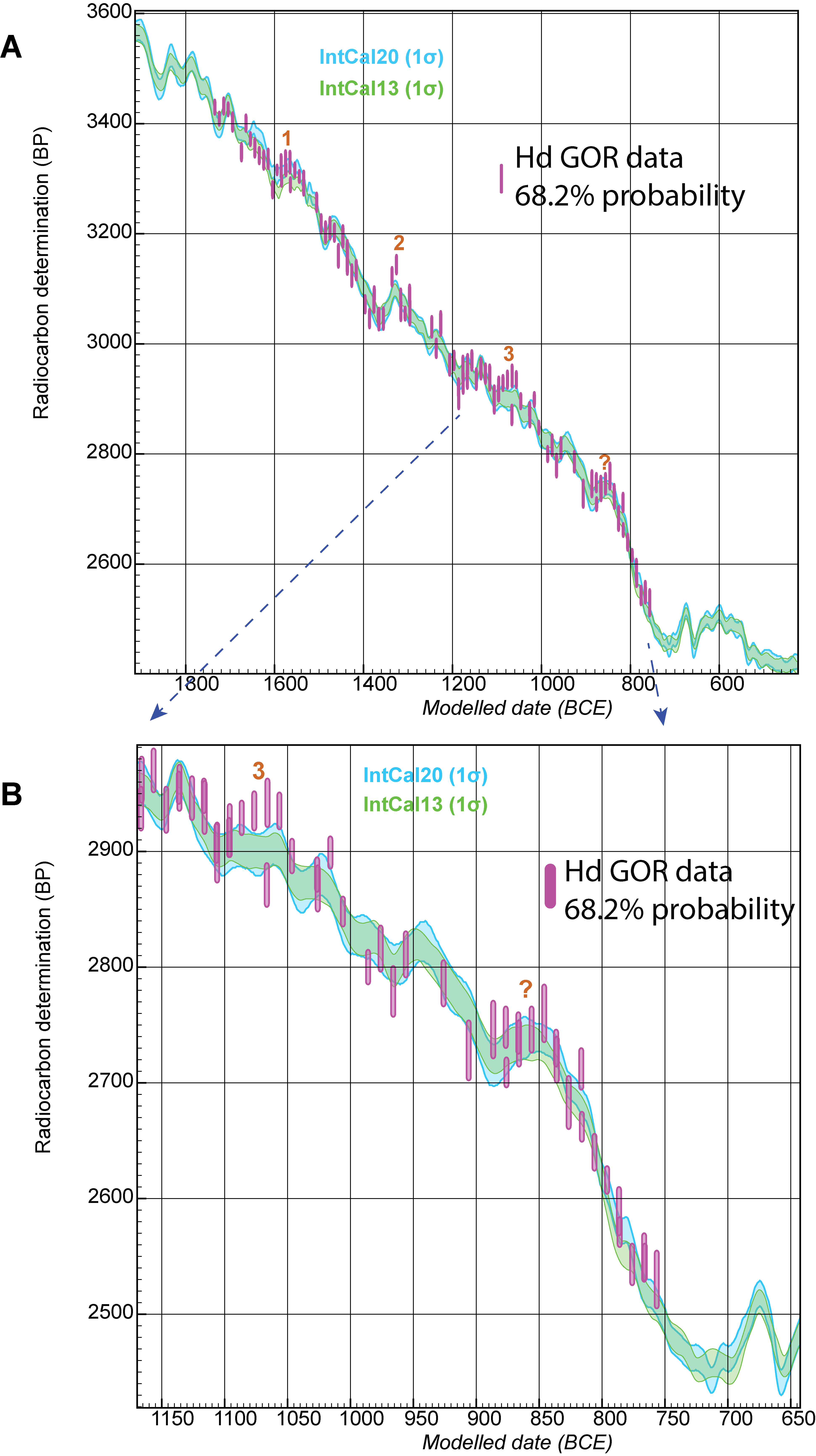

Supplement: S4 Fig — The IntCal13 calibration curve [134] is shown for comparison. The labels indicate: 1. a region in the 16th century BCE where previously there was an offset between the Hd GOR dataset and IntCal13 [180] but which is now largely removed with the revised IntCal20 dataset; 2. and 3. two regions (reversals and/or plateaus in the calibration curve) where there appear to be positive offsets between the Hd GOR data and IntCal20; and? another reversal and plateau where there is perhaps a small difference between the Hd GOR dataset and IntCal20. A. shows overall comparison, B. shows detail for the mid-12th to 8th centuries BCE. (TIF) [file pone.0240799.s007.tif]
